# PIWIL2 drives stroke progression via modulation of NF-κB signaling

**DOI:** 10.1016/j.omtn.2026.103037

**Published:** 2026-07-24

**Authors:** Rohan Mahesh Patil, Giusy Laudati, Natascia Guida, Silvia Ruggiero, Noemi Di Muraglia, Serenella Anzilotti, Julian Lanthaler, Luigi Coppola, Luigi Formisano, Lucio Annunziato, Elga Esposito, Giuseppe Pignataro

**Affiliations:** 1 Division of Pharmacology, Department of Neurosciences, Reproductive Sciences and Odontostomatology, University of Naples Federico II, 80131 Naples, Italy; 2 Department for the Promotion of Human Sciences and Quality of Life, San Raffaele University, 00166 Rome, Italy; 3 IRCCS SDN Naples, Via Gianturco 113 80143 Naples, Italy; 4 Neuroprotection Research Laboratories, Departments of Radiology and Neurology, Massachusetts General Hospital, Harvard Medical School, Charlestown, MA, USA

**Keywords:** MT: non-coding RNAs, PIWIL, brain inflammation, ischemic stroke, blood brain barrier, systemic role, RNA binding protein, chemokine response, NF-kB

## Abstract

PIWILs are RNA-binding proteins whose role in ischemic stroke remains poorly defined. Here, we investigated the contribution of PIWILs to post-ischemic neuroinflammation by using a mouse model of 1 h transient middle cerebral artery occlusion (tMCAo), followed by 6 or 24 h of reperfusion. Brain tissues, blood, and peripheral organs were collected to assess PIWIL expression, inflammatory responses, and brain damage. PIWIL1 and PIWIL2 were significantly upregulated in the cortex and striatum at 24 h post-ischemia, with PIWIL2 also increased in blood and peripheral tissues. Immunofluorescence analyses revealed cell-type-specific localization of PIWILs in neurons, astrocytes, and endothelial cells. Notably, siRNA-mediated silencing of PIWIL2 significantly reduced infarct volume. PIWIL2 silencing also attenuated NF-κB-p65 and phospho-IκBα levels in the peri-infarct cortex but not in the striatum, indicating region-specific modulation of NF-κB signaling and differentially reprogrammed systemic chemokine profiles, with selective upregulation of CCL3, CCL5, and CXCL13 and downregulation of CCL2, CCL17, CCL20, and CXCL1. Mechanistically, PIWIL2 interacted with IKKα and promoted activation of the NF-κB pathway, leading to RelA- and RelB-dependent transcriptional regulation of pro-inflammatory chemokines. Together, these findings identify PIWIL2 as an upstream regulator of NF-κB-dependent neuroinflammation that exacerbates ischemic brain injury and suggest PIWIL2 as a promising therapeutic target targeting post-stroke inflammatory damage.

## Graphical abstract

**Figure:**
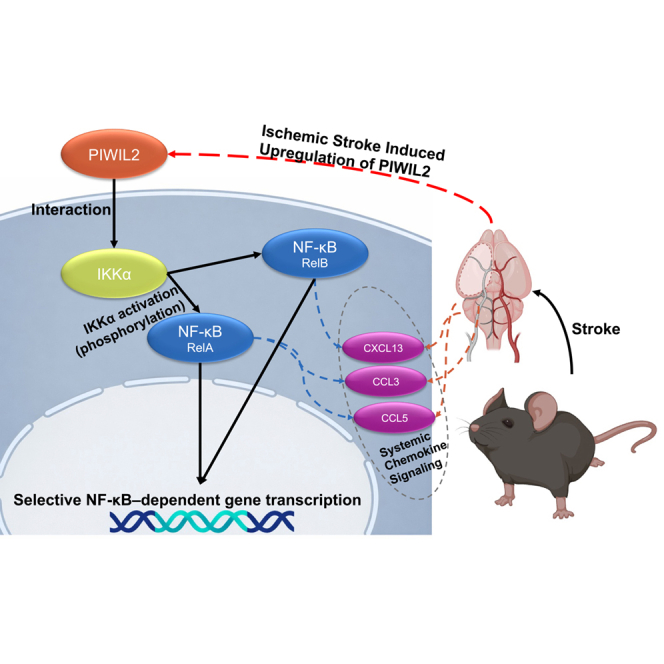

## Teaser

Pignataro and colleagues demonstrate that PIWIL2, a PIWI-family RNA-binding protein, drives post-ischemic neuroinflammation via IKKα/NF-κB signaling in a mouse stroke model. siRNA-mediated PIWIL2 silencing administered up to 3 h after ischemia onset significantly reduces infarct volume and reprograms systemic chemokine profiles, identifying PIWIL2 as a promising post-stroke therapeutic target.

## Introduction

Stroke is one of the leading causes of mortality and long-term disability worldwide, primarily resulting from the reduction of blood supply to the brain, which leads to ischemic injury and inflammation.^1^^,^^2^ The inflammatory response following stroke plays a crucial role in the progression of brain damage, particularly in the post-ischemic phase.^3^ Understanding the molecular mechanisms that regulate this inflammatory response is key to identifying therapeutic targets that can mitigate stroke-induced neuronal inflammation.

PIWI-like (PIWIL) proteins, originally characterized for their role in RNA silencing and genome regulation in the germ line, have recently gained attention for their involvement in several cellular processes in somatic tissues, including gene expression regulation, stem cell maintenance, and immune and inflammatory response modulation.^4^^,^^5^^,^^6^^,^^7^^,^^8^ In the context of stroke, emerging evidence suggests that PIWIL proteins and their corresponding messenger RNAs (mRNAs) may contribute to neuroinflammatory processes and stress response.^6^^,^^9^^,^^10^^,^^11^ However, the precise mechanisms by which PIWIL proteins contribute to the inflammatory cascade following brain ischemic injury remain unclear. Among PIWIL family members, PIWIL2 has been specifically implicated in somatic inflammatory regulation through direct interaction with the inhibitor of κB kinase (IKK) complex, promoting IKK phosphorylation, IκBα degradation, and subsequent nuclear factor-kappa B (NF-κB) nuclear translocation in esophageal cancer cells.^12^ NF-κB is a master regulator of post-ischemic neuroinflammation, driving transcription of pro-inflammatory cytokines, chemokines, and cell adhesion molecules that amplify secondary brain damage following stroke. Based on its unique systemic upregulation in blood and peripheral organs in addition to brain tissue and its established role in IKK/NF-κB signaling in somatic cells, PIWIL2 was prioritized for mechanistic investigation in the present study.

This study investigates the role of PIWIL mRNA and protein expression in a mouse model of stroke, with a particular focus on the pro-inflammatory response. By silencing PIWIL proteins through gene knockdown techniques, we aim to elucidate how the reduction of PIWIL activity affects the post-stroke inflammatory process. We hypothesized that PIWIL2 promotes neuroinflammatory signaling and exacerbates ischemic injury, and that its silencing would confer neuroprotection. The findings of this study provide new mechanistic insight into PIWIL2-dependent regulation of stroke progression and identify PIWIL2 as a potential target for modulating ischemic brain damage.

## Results

### Focal cerebral ischemia regulates PIWIL1 and PIWIL2 mRNA and protein expression

**Figure 1:**
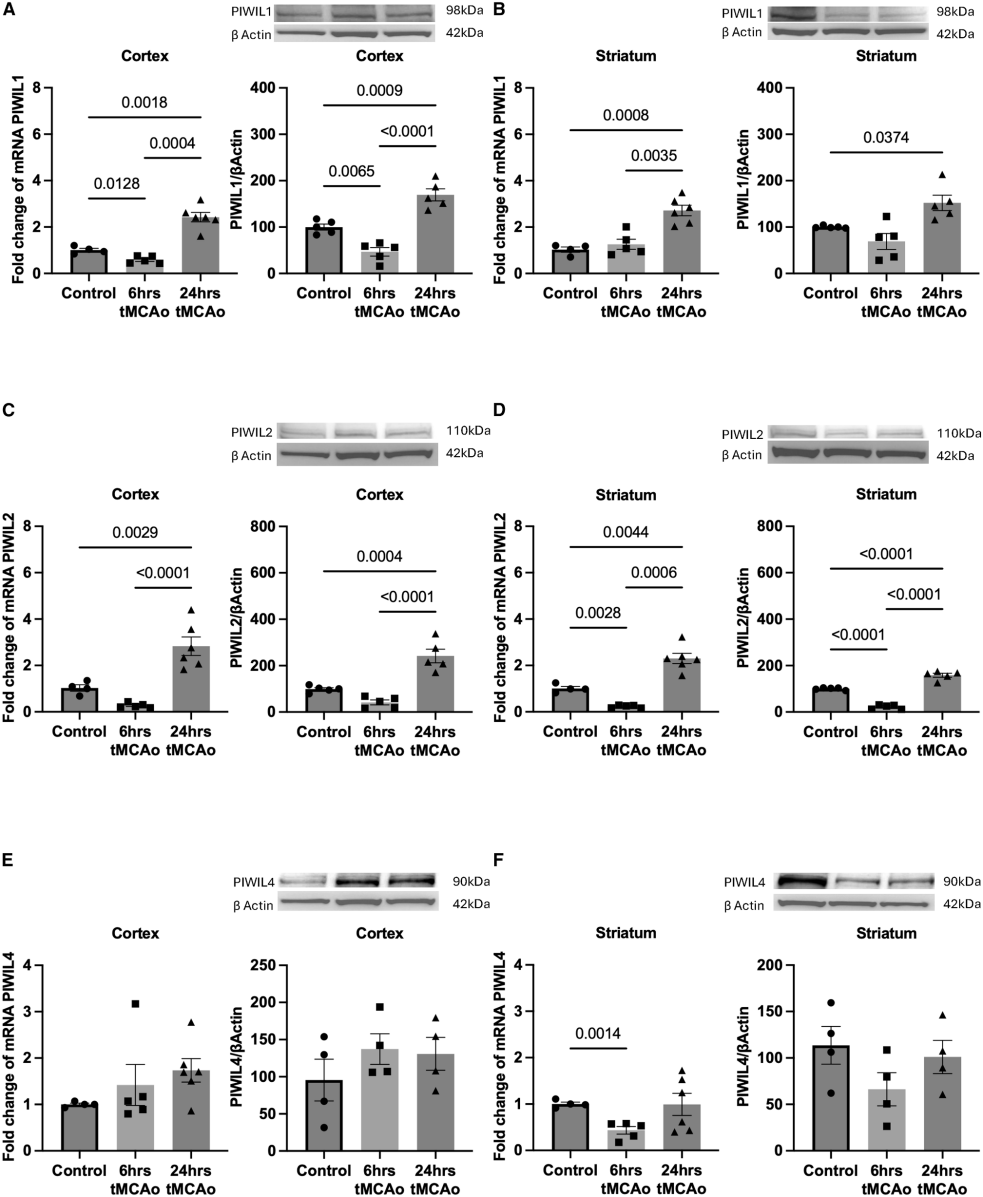
Expression level of PIWIL in the cortex and Striatum following tMCAo mRNA and protein expression of PIWIL1 (A and B), PIWIL2 (C and D), and PIWIL4 (E and F). Cortex levels are depicted in (A), (C), and (E) and striatum levels in (B), (D), and (F). Data are presented as fold change relative to control animals. mRNA expression was normalized to GAPDH, and protein expression was normalized to β-actin. Representative western blots are shown above the corresponding quantifications. Each bar represents mean ± SEM (*n* = 4–6 per group). mRNA data were analyzed using Brown-Forsythe and Welch ANOVA followed by Dunnett’s T3 post hoc test. Protein data were analyzed using one-way ANOVA followed by Tukey’s post hoc test.

To investigate the role of the PIWIL following ischemic stroke, we assessed both mRNA and protein expression of PIWIL1, PIWIL2, and PIWIL4 in the cerebral cortex and striatum of ischemic mice. At 24 h (Figures 1A–1D) post-stroke, PIWIL1 and PIWIL2 mRNA were significantly upregulated in both brain regions. This upregulation in the protein levels of PIWIL1 and PIWL2 was similar, indicating active translation and supporting a role for PIWIL proteins in post-ischemic inflammatory signaling and tissue responses during the post-stroke period. In contrast (Figures 1A and 1C), at 6 h post-stroke, PIWIL1 and PIWIL2 mRNAs were downregulated in the cortex, with proteins similarly reduced.

### PIWIL proteins presence in the neuronal cells in cortex and striatum

Given the observed differential expression of PIWIL proteins in both the cortex and the striatum at the mRNA and protein levels following ischemic stroke, we performed an immunofluorescence analysis to define their cell-type-specific localization under ischemic conditions. Analyses were conducted at 24 h post-ischemia in peri-infarct cortical and striatal regions, where cellular morphology remains preserved. Low-magnification images (40×) were used to provide anatomical context, while high-magnification confocal images (63× oil immersion) enabled assessment of intracellular localization and co-localization of PIWIL proteins within neurons, astrocytes, and endothelial cells.

**Figure 2:**
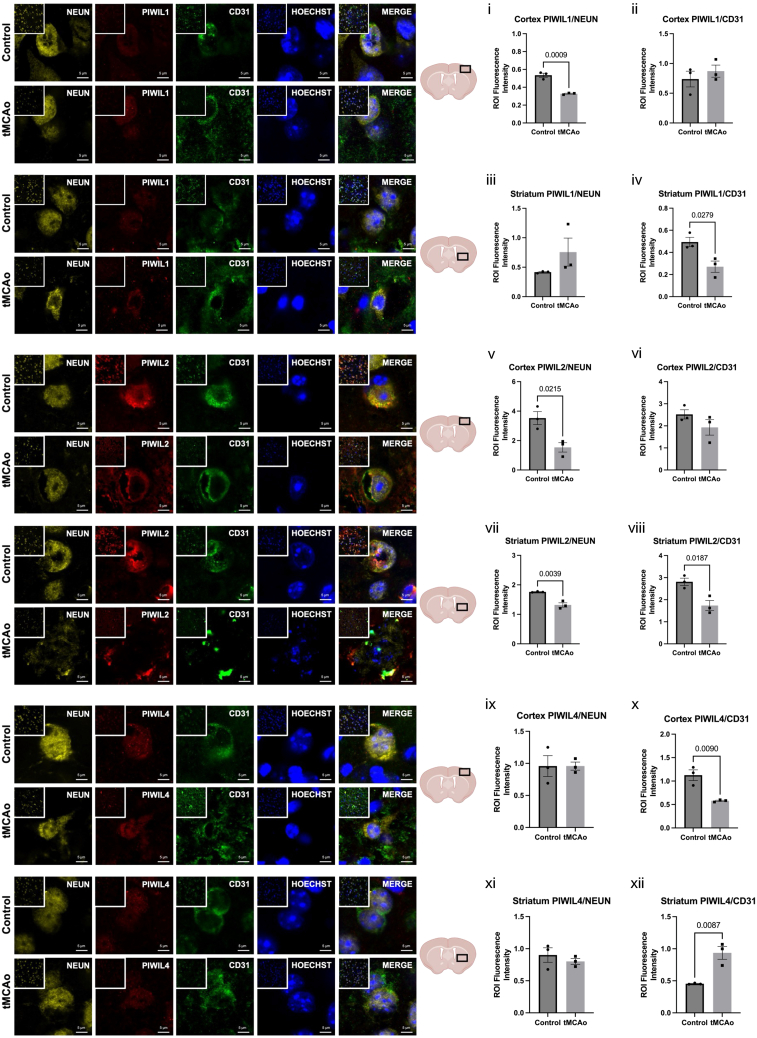
Differential expression of PIWIL1, PIWIL2, and PIWIL4 in endothelial cells (CD31) following tMCAo Immunofluorescence staining demonstrates the co-localization of PIWIL proteins (red) with neuronal (NeuN, yellow) and endothelial (CD31, green) markers in the cortex and striatum of control and tMCAo mice. Hoechst (blue) was used for nuclear visualization. Merged images display the overall expression and cellular localization. Representative images for PIWIL1, PIWIL2, and PIWIL4 are shown. Quantification of region of interest (ROI) intensity for PIWIL1/NeuN (i and iii), PIWIL1/CD31 (ii and iv), PIWIL2/NeuN (v and vii), PIWIL2/CD31 (vi and viii), PIWIL4/NeuN (ix and xi), and PIWIL4/CD31 (x and xii) demonstrates changes in protein expression in the cortex and striatum following tMCAo. Data are presented as mean ± SEM (*n* = 3 per group). Low-magnification overview images (40×) are shown as insets to provide regional context, while high-magnification confocal images (63× oil immersion) are used to assess cell-type-specific localization and intracellular co-localization of PIWIL proteins with NeuN and CD31. Statistical significance was determined by unpaired *t* test; ∗*p* < 0.05. Scale bars are indicated in each image.

**Figure 3:**
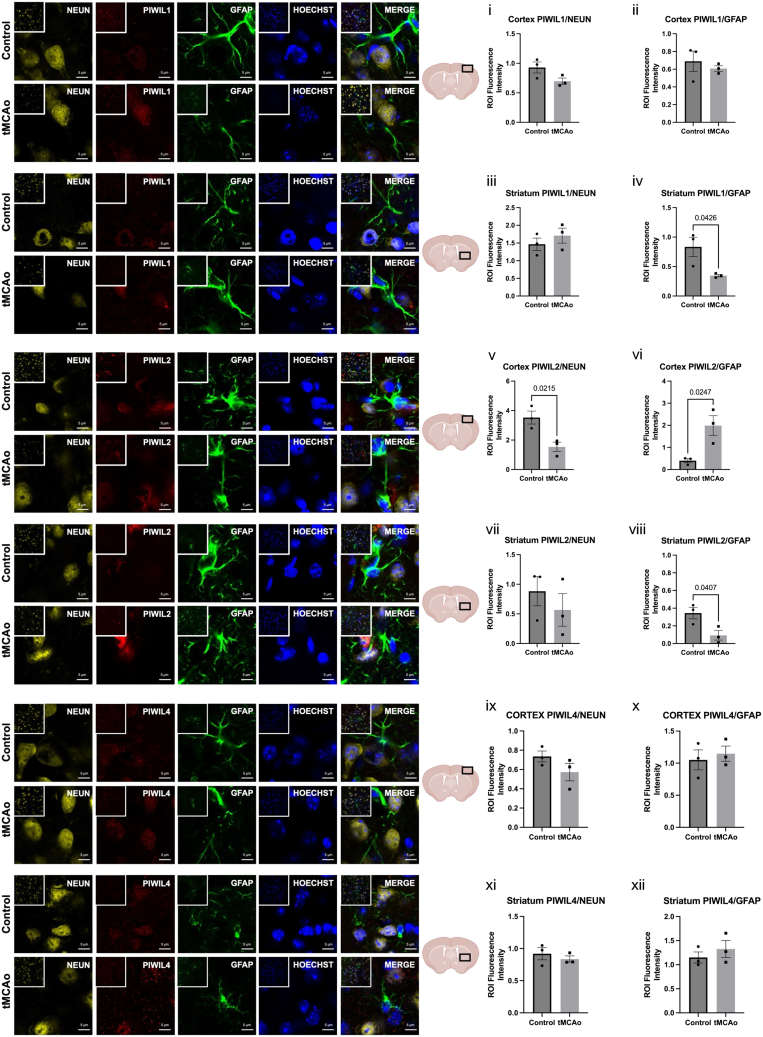
Differential expression of PIWIL1, PIWIL2, and PIWIL4 in astrocytes cells (GFAP) in cortical and striatal regions after tMCAo Shown are representative images for PIWIL1, PIWIL2, and PIWIL4 (red) co-stained with NeuN (yellow) and GFAP (green) and counterstained with Hoechst (blue) for nuclear visualization. Merged images display the overall expression and cellular localization. Quantification of region of interest (ROI) intensity for PIWIL1/NeuN (i and iii), PIWIL1/GFAP (ii and iv), PIWIL2/NeuN (v and vii), PIWIL2/GFAP (vi and viii), PIWIL4/NeuN (ix and xi), and PIWIL4/GFAP (x and xii) demonstrates changes in protein expression in the cortex and striatum following tMCAo. Low-magnification overview images (40×) are shown as insets to provide regional context, while high-magnification confocal images (63× oil immersion) are used to assess cell-type-specific localization and intracellular co-localization of PIWIL proteins with NeuN and GFAP. Data are presented as mean ± SEM (*n* = 3 per group). Statistical significance was determined by unpaired *t* test; ∗*p* < 0.05, ∗∗*p* < 0.01. Scale bars are indicated in each image.

Our findings demonstrated significant colocalization of PIWIL1, PIWIL2, and PIWIL4 with neuronal cells (NeuN), astrocytes (glial fibrillary acidic protein [GFAP]), and endothelial cells (CD31). While the fluorescence intensity was comparable across groups, there were notable differences in colocalization patterns, particularly with GFAP and CD31, which consistently showed lower colocalization compared to NeuN (Figures 2 and 3).

PIWIL1 protein expression was reduced in both CD31 and GFAP positive cells (Figures 2 iv and 3 iv). In contrast, PIWIL2 expression was increased in GFAP positive cells in the striatum but decreased in CD31 positive cells (Figures 3 vi and 2 viii, respectively). Most notably, PIWIL4 showed a distinct expression pattern in CD31 and NeuN positive cells, differing between the cortex and striatum (Figure 2 x and xii).

### Alteration of PIWIL RNA as post-ischemic distant tissue damage

**Figure 4:**
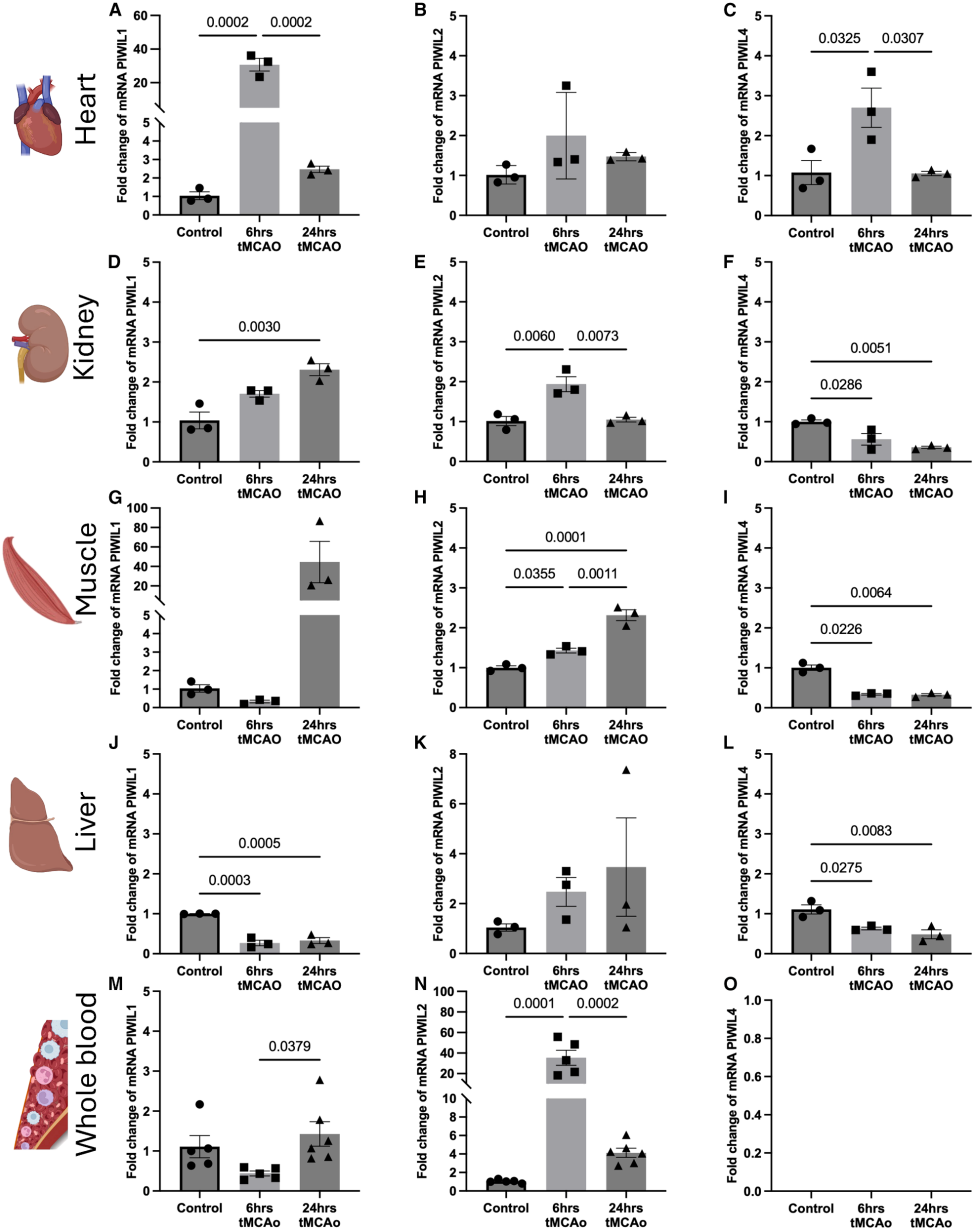
Tissue-specific mRNA expression of PIWIL1, PIWIL2, and PIWIL4 in various organs following tMCAo Quantitative real-time PCR analysis showing the fold change of mRNA levels of PIWIL1 (A, D, G, J, and M), PIWIL2 (B, E, H, K, and N), and PIWIL4 (C, F, I, L, and O) in the heart (A–C), kidney (D–F), liver (J–L), skeletal muscle (G–I), and whole blood (M–O) of control mice and mice at 6 and 24 h after tMCAo. Data are normalized to GAPDH and expressed relative to control animals. Each bar represents mean ± SEM (*n* = 3 per group). Statistical significance was determined by one-way ANOVA followed by Tukey’s post hoc test.

Following a stroke, it has been well demonstrated that several body systems are affected, with key functions in distant organs disrupted due to insufficient signaling responses and inflammatory processes. The heart, liver, kidneys, and skeletal muscles are particularly vulnerable to these post-stroke effects, as the body attempts to adapt to the damage caused by ischemia (Figure 4). Significant changes in gene expression occur at critical time points 6 and 24 h after the stroke, which reflect the evolving systemic response to the injury.

PIWIL1 expression was considerably elevated in the heart at 6 h post-stroke (Figure 4A), while PIWIL2 showed no significant change (Figure 4B), and PIWIL4 was significantly upregulated at 6 h (Figure 4C). In the kidney, PIWIL1 was significantly upregulated at 24 h post-stroke (Figure 4D), while PIWIL2 was transiently elevated at 6 h but returned to baseline levels by 24 h (Figure 4E). In contrast, PIWIL4 was significantly downregulated at both time points (Figure 4F). In skeletal muscle, PIWIL2 was significantly increased at both time points (Figure 4H), while PIWIL4 was consistently downregulated (Figure 4I). In the liver, PIWIL1 and PIWIL4 were downregulated at both 6 and 24 h (Figures 4J and 4L), with PIWIL2 showing no significant change (Figure 4K).

### PIWIL2 alteration in the whole blood mRNA expression

Analysis of whole-blood RNA indicated that PIWIL2 expression was considerably increased at 6 h post-stroke, followed by a decrease at 24 h (Figure 4N); nonetheless, it was significantly higher than in the control group. PIWIL1 expression remained constant, indicating a trend, although not statistically significant (Figure 4M). Surprisingly, PIWIL4 expression was absent in the blood (Figure 4O).

### Pro-inflammatory response following stroke

Since it is well known that PIWIL proteins are involved in controlling the inflammation in the brain during stroke, and to better understand the function, we analyzed all the pro-inflammatory markers in the plasma samples from all the animal included in the study.

**Figure 5:**
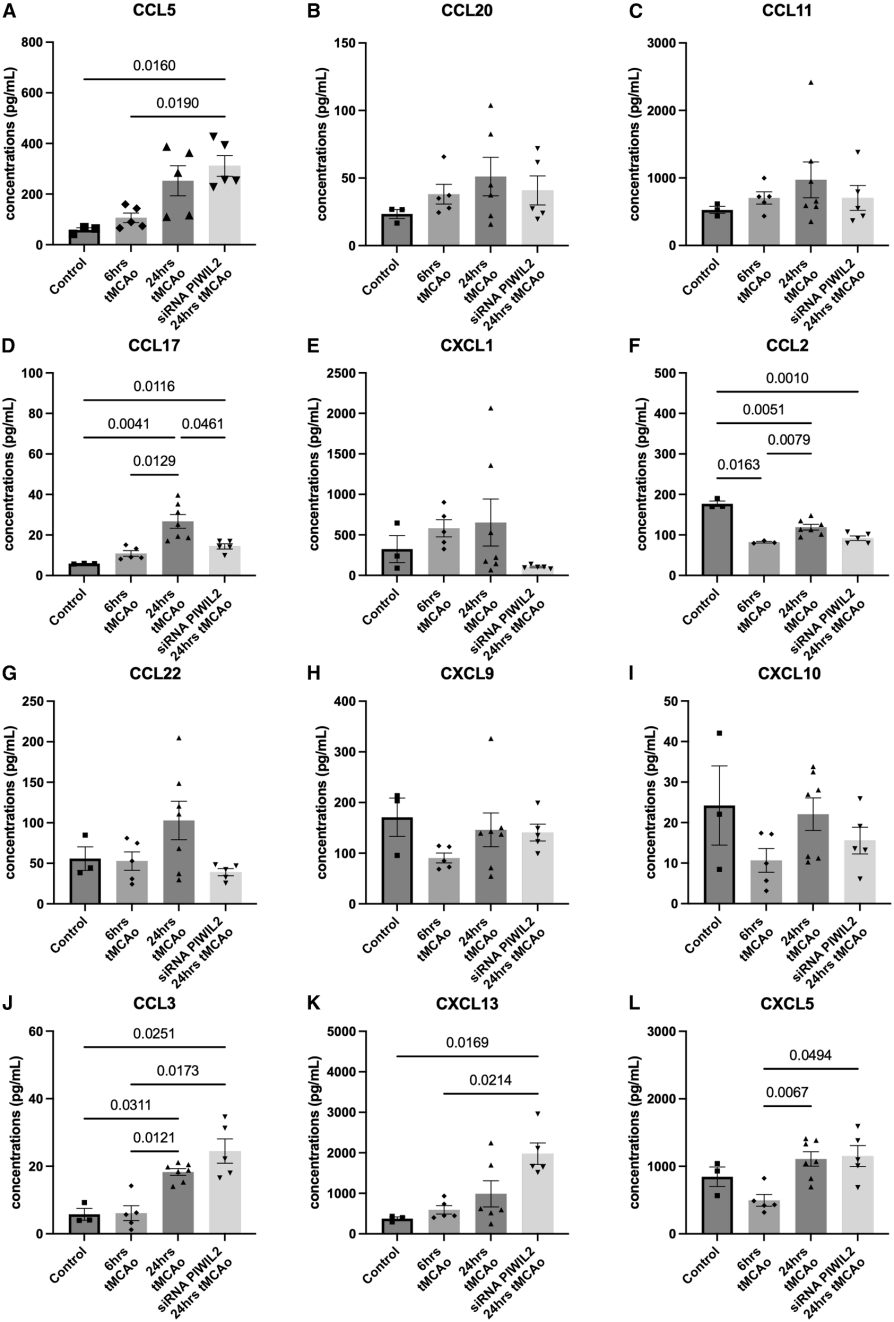
Effect of PIWIL2 knockdown on circulating chemokine levels after tMCAo Concentrations (pg/mL) of various chemokines CCL5 (A), CCL20 (B), CCL11 (C), CCL17 (D), CXCL1 (E), CCL2 (F), CCL22 (G), CXCL9 (H), CXCL10 (I), CCL3 (J), CXCL13 (K), and CXCL5 (L) in the plasma of control mice, tMCAo mice at 6 h and 24 h post-ischemia, and tMCAo mice treated with PIWIL2 siRNA at 24 post-ischemia. Data are presented as mean ± SEM (*n* = 3–7 per group). Statistical significance was determined by Brown-Forsythe and Welch ANOVA followed by Dunnett’s T3 post hoc test

CCL2 (Figure 5F) expression was low at 6 h and 24 h post-stroke, as all the other markers, specially CCL3, CCL5, CCL13, CCL17, CCL20, and CXCL13, increased gradually in the samples from 6 h to 24 h. CCL22 was not altered at the6 h time point but was increased after 24 h. CXCL9 and CXCL10 were lowered at 6 h and returned to baseline levels by 24 h post-ischemia. CCL4 was not detected in the plasma samples.

### Silencing PIWIL2 reduces infarct volume, modulates NF-κB signaling, and reprograms systemic chemokine profiles

**Figure 6:**
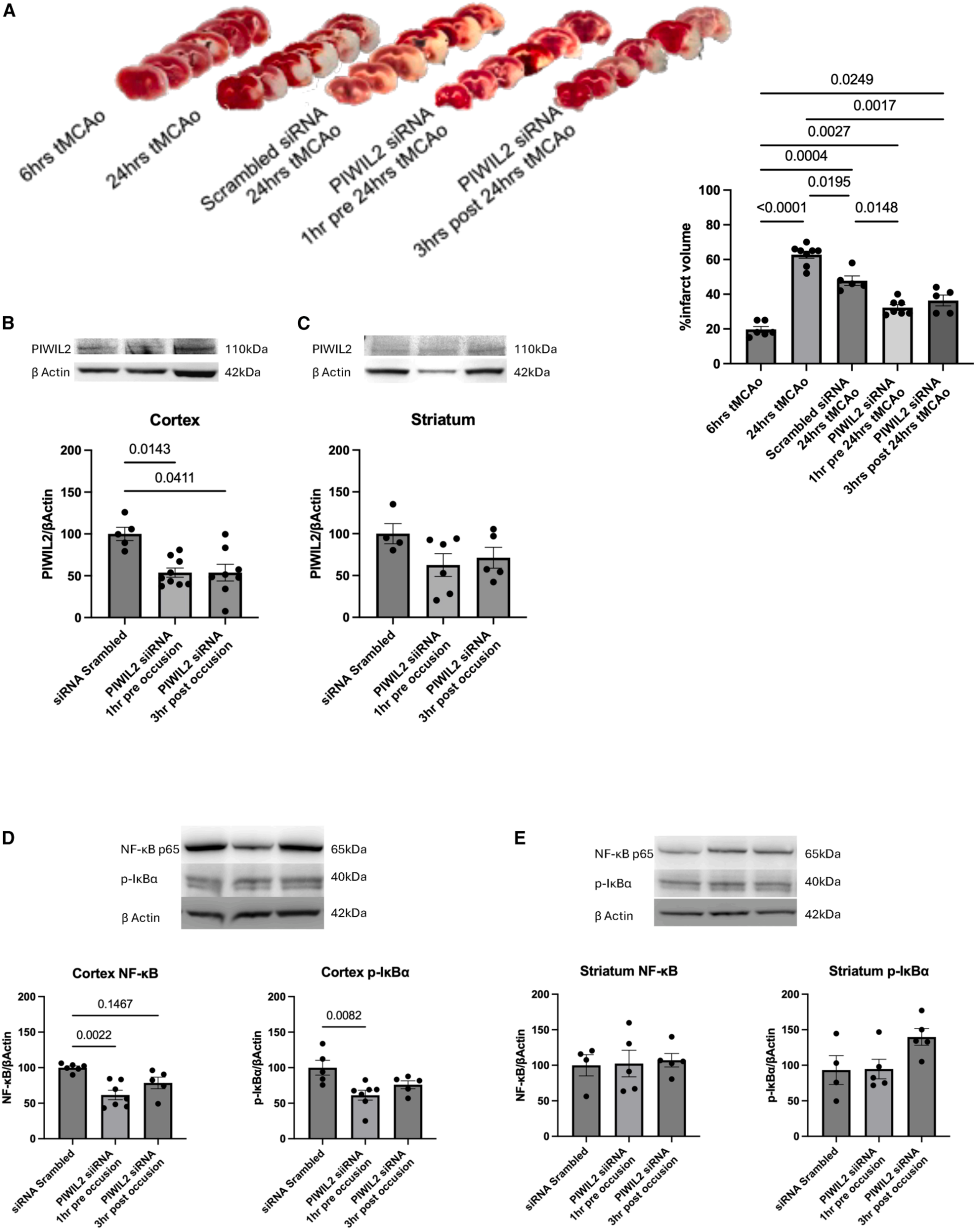
PIWIL2 silencing reduces infarct volume, attenuates NF-κB signaling and reprograms systemic chemokine profiles after tMCAo (A) Representative TTC-stained brain sections and infarct volume quantification (% ipsilateral hemisphere) in mice subjected to 6 h tMCAo, 24 h tMCAo, scrambled siRNA +24 h tMCAo, PIWIL2 siRNA administered 1 h pre-ischemia, and PIWIL2 siRNA administered 3 h post-ischemia. (B and C) Western blot analysis and quantification of PIWIL2 protein expression normalized to β-actin in the ipsilateral cortex (B) and striatum (C) from scrambled siRNA, PIWIL2 siRNA 1 h pre-occlusion, and PIWIL2 siRNA 3 h post-occlusion groups. (D) Representative Western blots and quantification of NF-κB p65 (65 kDa) and phospho-IκBα Ser32 (40 kDa), normalized to β-actin (42 kDa), in the ipsilateral cortex from scrambled siRNA, PIWIL2 siRNA 1 h pre-ischemia, and PIWIL2 siRNA 3 h post-ischemia groups. (E) Representative western blots and quantification of NF-κB p65 and phospho-IκBα Ser32 in the striatum from the same experimental groups, showing no significant changes. Data are presented as mean ± SEM (*n* = 3–8 per group). Infarct volume and chemokine data were analyzed using Brown-Forsythe and Welch ANOVA followed by Dunnett’s T3 post hoc test. Western blot data were analyzed using Kruskal-Wallis test followed by Dunn’s post hoc test. Exact *p* values are indicated above bars where significant.

To assess the translational relevance of PIWIL2 as a therapeutic target, siRNA was administered intracerebroventricularly (ICV) either 1 h before or 3 h after transient middle cerebral artery occlusion (tMCAo) onset, alongside a scrambled siRNA control group. Scrambled siRNA had no significant effect on infarct volume compared with untreated 24-h tMCAo animals, confirming that ICV delivery per se does not influence ischemic outcome (Figure 6A). In contrast, PIWIL2-specific siRNA administered either 1 h pre-ischemia or 3 h post-ischemia significantly reduced infarct volume compared with both the 24-h tMCAo and scrambled siRNA groups (Figure 6A), demonstrating that PIWIL2 silencing confers neuroprotection even when initiated after stroke onset. PIWIL2 protein expression was significantly reduced in the ipsilateral cortex following siRNA treatment (Figure 6B), with a reduction exceeding 40% compared with scrambled controls, confirming effective knockdown. A similar trend was observed in the striatum, although this did not reach statistical significance, likely reflecting greater tissue heterogeneity in the core ischemic region (Figure 6C).

Silencing PIWIL2 markedly altered the circulating chemokine profile (Figure 5). Specifically, 24 h post-ischemia PIWIL2 silencing resulted in a further increase in CCL3, CCL5, and CXCL13 levels in the plasma, whereas CCL11 and CCL22 returned to baseline. These chemokines are recognized mediators of leukocyte recruitment and secondary inflammatory damage, indicating that PIWIL2 silencing reprograms rather than uniformly suppresses inflammatory signaling.^13^^,^^14^^,^^15^ CCL2, CCL11, and CCL20 were reduced to the level of 6 h after stroke, lower than 24 h but not as that of control. CXCL1 showed a severe reduction and was lower than in the control samples as well. CXCL5 and CXCL9 levels were similar to 24 h stroke samples.

To further investigate whether PIWIL2 silencing modulates the NF-κB signaling pathway, western blot analyses of NF-κB p65 and phospho-IκBα (Ser32) were performed in ipsilateral cortex and striatum tissue from scrambled siRNA, PIWIL2 siRNA 1 h pre-ischemia, and PIWIL2 siRNA 3 h post-ischemia groups. In the ipsilateral cortex, PIWIL2 silencing significantly reduced NF-κB p65 protein levels compared with scrambled siRNA controls (*p* = 0.0022), with a similar trend observed at 3 h post-ischemia (Figure 6D). Consistent with this, phospho-IκBα (Ser32) levels were also significantly reduced following PIWIL2 knockdown at 1 h pre-ischemia (*p* = 0.0082), indicating attenuation of canonical NF-κB pathway activation (Figure 6D). In contrast, neither NF-κB p65 nor phospho-IκBα levels were significantly altered in the striatum following PIWIL2 silencing (Figure 6E), suggesting that PIWIL2-dependent NF-κB modulation is region-specific and predominantly occurs in the peri-infarct cortical tissue.

### PIWIL2 activates the IKK/NF-κB pathway and selectively regulates chemokine gene transcription following cerebral ischemia

**Figure 7:**
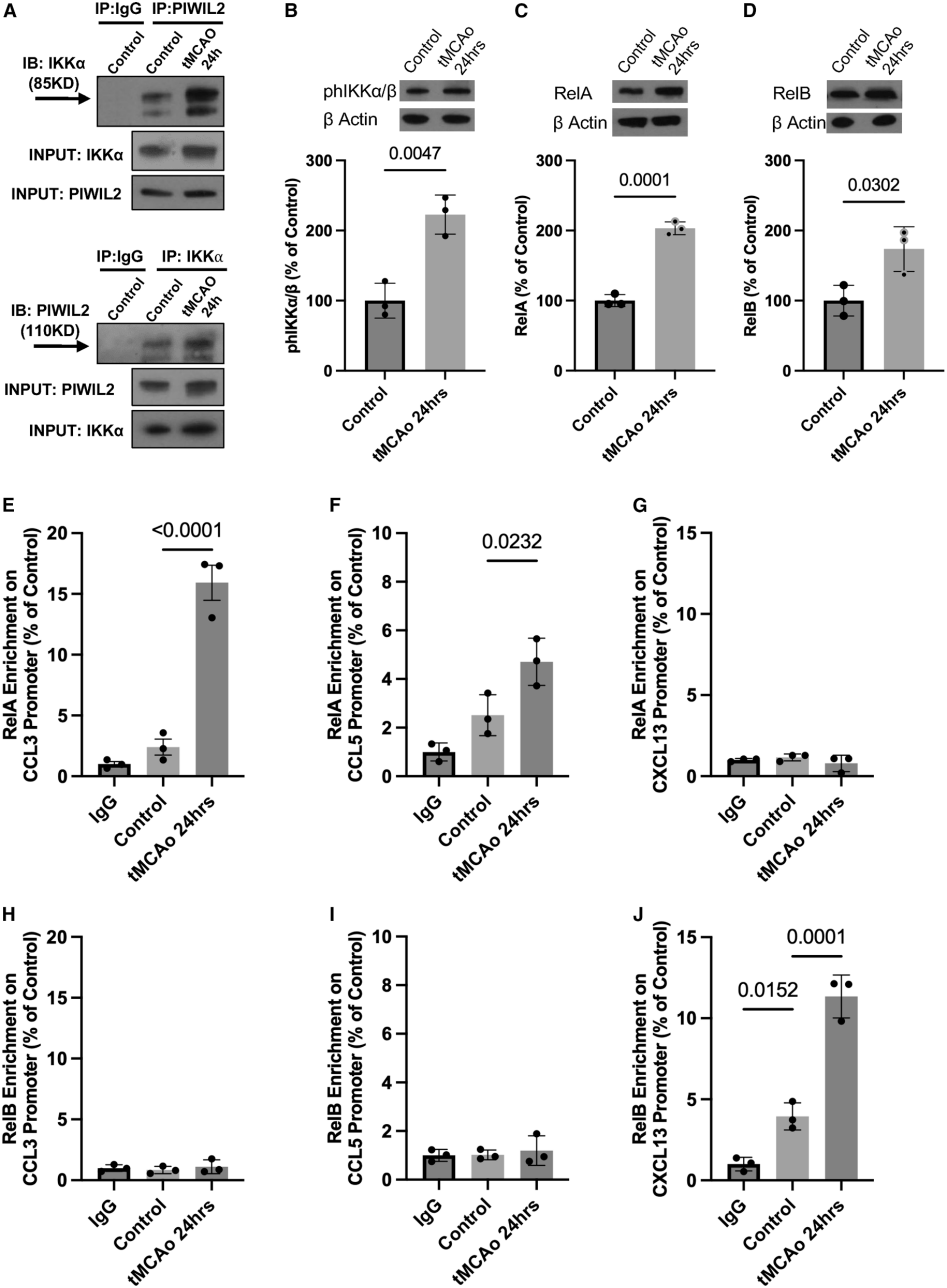
PIWIL2 activates RelA- and RelB-dependent transcriptional programs following cerebral ischemia (A) Co-immunoprecipitation (coIP) analysis showing the interaction between PIWIL2 and IKKα (top) and reciprocal immunoprecipitation of IKKα with PIWIL2 (bottom) in cortical lysates from naive (control) mice and mice subjected to transient middle cerebral artery occlusion followed by 24 h of reperfusion (tMCAo). Immunoprecipitation with IgG was used as a negative control. (B–D) Western blot analysis of phosphorylated IKKα/β (Ser176/180) (B), RelA (NF-κB p65) (C), and RelB (D) protein levels in cortical tissues from control and tMCAo mice. (E–G) Chromatin immunoprecipitation (ChIP) analysis of RelA binding to the CCL3 (E), CCL5 (F), and CXCL13 (G) promoter regions in cortical tissues from control and tMCAo mice. (H–J) ChIP analysis of RelB binding to the CCL3 (H), CCL5 (I), and CXCL13 (J) promoter regions in cortical tissues from Control and tMCAo mice. ChIP data were normalized to total input chromatin and expressed as percentage relative to controls. Data are presented as mean ± SEM (*n* = 3 independent biological replicates per group). ∗*p* < 0.05 vs. control, determined by unpaired two-tailed Student’s *t* test.

To elucidate the molecular mechanism through which PIWIL2 exacerbates ischemic brain injury and modulates inflammatory responses, we investigated the involvement of the canonical NF-κB signaling pathway in the ischemic cortex. Co-immunoprecipitation (coIP) experiments revealed a physical interaction between PIWIL2 and IKKα (inhibitor of κB kinase alpha) in ischemic cortex lysates from mice subjected to tMCAo followed by 24 h of reperfusion, whereas this interaction was negligible in naive mice (Figure 7A). Reciprocal immunoprecipitation by using an anti-IKKα antibody confirmed the specificity of this interaction, indicating that PIWIL2 directly associates with upstream components of the NF-κB signaling cascade following cerebral ischemia. Consistent with this interaction, western blot analysis demonstrated a significant increase in the phosphorylation of IKKα/β at Ser176/180 in ischemic cortex compared with naive mice (Figure 7B), indicative of NF-κB pathway activation. This was followed by a marked upregulation of the NF-κB subunits RelA (p65) and RelB at 24 h after ischemia (Figures 7C and 7D), suggesting sustained activation of both canonical and non-canonical NF-κB signaling pathways. To determine whether PIWIL2-dependent NF-κB activation translated into transcriptional regulation of inflammatory chemokines, chromatin immunoprecipitation (ChIP) assays were performed in cortical tissue. RelA showed a significant enrichment at the promoter regions of CCL3 and CCL5 in ischemic mice compared with controls, whereas no significant binding was detected at the CXCL13 promoter (Figures 7E–7G). In contrast, RelB displayed selective enrichment at the CXCL13 promoter, with no significant binding to CCL3 or CCL5 regulatory regions (Figures 7H–7J). These findings demonstrate that PIWIL2 promotes ischemia-induced neuroinflammation by engaging the IKK/NF-κB signaling axis and driving distinct RelA- and RelB-dependent transcriptional programs. The selective recruitment of NF-κB subunits to specific chemokine promoters provides a mechanistic explanation for the differential modulation of circulating and brain inflammatory mediators observed following PIWIL2 silencing (Figure 5).

## Discussion

This study reveals a previously unrecognized role for PIWIL proteins in modulating neuroinflammation, especially in the context of stroke. While the presence and function of PIWIL proteins in the brain are not well understood, emerging evidence points to their involvement in critical neurological processes such as neurogenesis, astrogliosis, and inflammation.^6^^,^^8^ This research is the first to directly examine the role of PIWIL proteins in stroke pathophysiology, offering new direction into their potential impact in brain pathophysiology.^6^^,^^8^

PIWIL proteins are mainly known for their role in gene silencing through interactions with PIWI-interacting RNAs (piRNAs), which help regulate gene expression and maintain genome integrity in germ cells.^9^^,^^16^^,^^17^^,^^18^ However, their functions in the brain appear to extend beyond piRNA interactions.

PIWIL1 and PIWIL2 exhibit complex transcriptional and translational dynamics in response to cerebral ischemia, with early downregulation at 6 h followed by upregulation at 24 h, indicating a role in the brain’s delayed response to ischemic injury (Figure 1). While PIWIL4 mRNA showed variability, its protein levels remained unaffected, pointing at a more selective or delayed role in stroke recovery. The upregulation of PIWIL2 in the bloodstream further highlights the systemic nature of stroke’s impact, suggesting that PIWIL2 serve as a potential biomarker or mediator in both neural and peripheral responses to ischemic injury as observed in distant organs.^6^^,^^17^^,^^19^^,^^20^

In the heart, both PIWIL1 and PIWIL4 were significantly upregulated 6 h post-stroke, indicating an early transcriptional response to ischemic stress and inflammation, potentially driven by the need to protect cardiomyocytes and modulate inflammation via RNA-mediated gene silencing mechanisms.^19^^,^^21^ By 24 h, PIWIL1 remained upregulated, with PIWIL2 also showing a marked increase, suggesting that these proteins may support longer-term adaptive and reparative mechanisms, such as controlling inflammation and apoptosis.^17^ In the kidneys, PIWIL1 and PIWIL2 were upregulated at 6 h, likely reflecting their role in managing systemic imbalances, while PIWIL4 downregulation suggests that it may act as a regulator of these responses, possibly limiting excessive inflammation or damage.^22^^,^^23^^,^^24^ In the liver, PIWIL1 and PIWIL4 were consistently downregulated at 6 and 24 h, possibly because of the metabolic adaptation during ischemic stress, although early PIWIL2 upregulation hints at a transient role in acute stress response.^20^^,^^25^^,^^26^ In skeletal muscle, PIWIL2 was upregulated at 6 and 24 h, suggesting a role in muscle adaptation and inflammation management, while PIWIL1 was only upregulated at 6 h, and PIWIL4 was consistently downregulated, indicating that PIWIL1 and PIWIL2 may be involved in early protective responses, while PIWIL4 plays a minimal role in muscle recovery, potentially acting as a suppressor of prolonged inflammation.^27^^,^^28^

PIWIL2 and PIWIL4 exhibited distinct colocalization with astrocytes, as evidenced by their association with GFAP (Figures 2 and 3). This suggests that these proteins are not only implicated in neuronal recovery but also involved in astrocytic functions under ischemic conditions. Astrocytes are known to play a pivotal role in maintaining the structural integrity of the brain, regulating inflammation, and supporting neuronal repair after injury. Therefore, the decreased expression in these cells could indicate their role in glial response to ischemic stress, potentially contributing to the regulation of neuroinflammation and gliosis.^8^ Although Iba1 staining was performed as part of our immunofluorescence panel, no significant colocalization of PIWIL proteins with Iba1-positive microglia was detected at 24 h post-ischemia in either cortex or striatum. Given that microglial activation is a progressive process that intensifies beyond the acute 24-h window examined here, future studies should investigate PIWIL2 expression in microglia at later post-ischemic time points, where its contribution to sustained neuroinflammation may be more prominent.

Interestingly, PIWIL4 expression was consistently reduced in the striatum (the core ischemic region) across all cell types compared with the control, suggesting a potential role in the peri-infarct region. PIWIL4 was primarily localized in the nuclei of various cell types, indicating its involvement in nuclear processes such as transcriptional regulation, RNA silencing, or chromatin remodeling, rather than in cytoplasmic or membrane-related activities after stroke.^19^^,^^29^ Unlike PIWIL1 and PIWIL2, PIWIL4’s mRNA and protein levels remained stable under both control and ischemic conditions, with no significant changes observed following stroke. Additionally, PIWIL4 was absent in whole blood, suggesting its function is likely confined to brain cellular processes, with no involvement in systemic circulation post-ischemic injury.

Crucially, our study demonstrates that PIWIL2 actively drives inflammatory signaling following ischemic injury. PIWIL2 silencing significantly reduced infarct volume and profoundly altered circulating chemokine profiles. To elucidate the underlying mechanism, we identified a direct interaction between PIWIL2 and IKKα, accompanied by increased phosphorylation of IKKα/β and accumulation of the NF-κB subunits RelA and RelB. These findings provide the first direct evidence in the ischemic brain that PIWIL2 acts upstream of NF-κB activation, extending previous observations made in cancer contexts to post-ischemic neuroinflammation,^12^ and indicate that PIWIL2 promotes inflammatory signal transduction rather than merely reflecting tissue injury.

ChIP analyses further revealed that PIWIL2-dependent NF-κB activation leads to selective transcriptional regulation of chemokines. RelA preferentially bound CCL3 and CCL5 promotors, key mediators of leukocyte recruitment and secondary inflammatory damage,^13^^,^^14^ whereas RelB selectively targeted the CXCL13 promoter, which is associated with immune cell organization and sustained, detrimental inflammatory signaling.^15^ This selective promoter occupancy may provide a mechanistic explanation for the differential chemokine responses observed following PIWIL2 modulation and supports a model in which PIWIL2 orchestrates distinct inflammatory programs through canonical and non-canonical NF-κB pathways.

Notably, PIWIL2 silencing significantly reduced circulating CCL17 levels (Figure 5D). CCL17 has been reported to exert neuroprotective effects through CCR4-dependent signaling in experimental hemorrhagic stroke models, including intracerebral and subarachnoid hemorrhage.^30^^,^^31^ Whether PIWIL2-dependent modulation of CCL17 plays a similar neuroprotective role in the ischemic context remains an open and important question for future investigation.

Overall, these findings identify PIWIL2 as a previously unrecognized upstream regulator of NF-κB-dependent neuroinflammation that exacerbates ischemic brain injury. By modulating both central and systemic inflammatory responses, PIWIL2 emerges as a promising therapeutic target for limiting post-ischemic inflammation and reducing infarct progression.

Nevertheless, further studies are warranted to elucidate the precise molecular interaction domains between PIWIL2 and IKKα^12^ and the contribution of piRNA-dependent versus piRNA-independent pathways. Notably, post-ischemic PIWIL2 silencing at 3 h after stroke onset significantly reduced infarct volume, providing proof-of-concept for the translational potential of this approach. Future work should assess clinically relevant PIWIL2 targeting strategies in larger preclinical settings, and PIWIL2 genetic knockout models^32^ remain an important future priority.

## Materials and methods

### Animals

Eight-to ten-week-old male C57 mice (22–24 g, Charles River) were housed in individually ventilated cages under diurnal lighting conditions (12-h light/dark cycles) in a temperature- and humidity-controlled room. To allow proper acclimatization, surgical procedures were performed one week after the animals’ arrival at the facility. All animal handling, care, and experimental procedures were conducted in accordance with the ARRIVE guidelines and the Guide for the Care and Use of Laboratory Animals. The experimental protocol was approved by the Animal Care Committee of the “Federico II” University of Naples, and experiments adhered to international guidelines for animal research. Animals that did not show at least a 70% reduction in cerebral blood flow or that died following ischemia induction (approximately 20% of the total mice) were excluded from the study. The remaining animals were randomly assigned to experimental groups. siRNA treatment was administered in a blinded fashion, and all data analyses were performed by a researcher blinded to the treatment conditions to ensure unbiased results.

### Transient occlusion of the MCA

Transient focal ischemia was induced, as previously described.^2^ In brief, occlusion of the middle cerebral artery (MCA) was performed in male mice anesthetized using a mixture of oxygen and sevoflurane at 3.5% (medical oxygen concentrator LFY-I-5A). Throughout the surgical procedure, animals were maintained under controlled anesthetic conditions, and body temperature was continuously monitored and maintained at physiological levels using a thermostatically controlled heating pad. A 6-O surgical monofilament nylon suture (Doccol, Sharon, MA) was inserted from the external carotid artery into the internal carotid artery and advanced into the circle of Willis up to the branching point of the MCA, thereby occluding the MCA for 1 h. Following occlusion, reperfusion for 6 and 24 h was performed.^33^^,^^34^

Animals were sacrificed with sevoflurane overdose 6 and 24 h after ischemia. The infarct volume was observed to confirm the occlusion. Organs (heart, liver, kidney, and skeletal muscle) and blood was extracted.

### Stereotaxic injection

Mice positioned on a stereotaxic frame, a 26G stainless steel guide cannula was implanted into the right lateral ventricle using the stereotaxic coordinates from the bregma: 0.3 mm caudal, 1 mm lateral, and 3 mm below the dura. PIWIL2-specific siRNA (Silencer Pre-designed siRNA, Ambion/Life Technologies, Part No. AM16708, siRNA ID: 181742; sense: 5′-CGAGAAGUGCCUCCUUUAGtt-3′; antisense: 5′-CUAAAGGAGGCACUUCUCGtt-3′) or scrambled control siRNA (Silencer Negative Control siRNA, Invitrogen) (5 μL, 5nM) were administered ICV either 1 h prior to tMCAo onset or 3 h following tMCAo onset. Knockdown efficiency was validated by western blot analysis of PIWIL2 protein levels in ipsilateral cortex and striatum (Figures 6B and 6C).

### Evaluation of the infarct volume

Mice were sacrificed after 6 or 24 h following siRNA administration (scrambled siRNA, PIWIL2 siRNA 1 h pre-ischemia, or PIWIL2 siRNA 3 h post-ischemia). The ischemic volume was evaluated by 2,3,5-triphenyl tetrazolium chloride (TTC) staining. The brains were cut into 1 mm coronal slices with a matrix. The total infarct volume, corrected for edema, was expressed as percentage of the volume of the hemisphere ipsilateral to the lesion.

### Quantitative PCR analysis

Total RNA was extracted from both brain tissues—specifically, slices from the ipsilateral cortex and striatum collected from naive and tMCAo-operated mice at 6 and 24 h post-surgery—and peripheral organs were extracted using the mirVana miRNA Isolation Kit, following the manufacturer’s protocol. For blood samples, RNA was isolated using the GeneJET Whole Blood RNA Purification Kit (Thermo Fisher Scientific), following the supplier’s protocol.

For RNA analysis, 200 ng of RNA was specifically reverse transcribed in the target cDNA, using High-Capacity cDNA Reverse Transcription Kit (Applied Biosystems) and TaqMan probes, following TaqMan assays protocol (25°C for 10 min, 37°C for 120 min, and 85°C for 5 min). Quantitative real-time PCR was performed with TaqMan Advanced Master Mix (Applied Biosystems) in a StepOnePlus (AB Applied Biosystems). cDNA samples were amplified simultaneously in triplicate in one assay run, following the protocol for TaqMan assays: 50°C for 2 min, 95°C for 10 min, 40 cycles of amplification of 95°C for 15 s, and 60°C for 1 min. All reactions were run in triplicate. Results were analyzed and exported with StepOnePlus System SDS software. TaqMan probes used are the following: PIWIL1 (assay ID: Mm00451649_m1), PIWIL2 (assay ID: Mm00502383_m1), PIWIL4 (assay ID: Mm01144775_m1), and GAPDH (assay ID: Mm99999915_g1).

### Western-blot

In the experimental setup, brain slices from the ipsilateral cortex and striatum were collected from the naive and tMCAo-operated mice at 6 and 24 h post-ischemia and ICV siRNA administration followed by ischemia of 24 h. Control samples were obtained from normal, untreated animals. The brain tissues were homogenized in a lysis buffer (50 mmol/L Tris-HCl, pH 7.5; 100 mmol/L NaCl; 1% Triton X-100) containing both protease and phosphatase inhibitors. The homogenates was kept on ice for 30 min followed by centrifuged at 12,000 ×*g* for 20 min at 4°C, and the resulting supernatants were collected for further analysis. Protein concentration was estimated using the Bradford reagent. Then, 20 μg of protein was mixed with NuPAGE LDS Sample Buffer (Thermo Fisher Scientific) and heated at 95°C for 5 min. Proteins were separated using NuPAGE 4–12% Bis-Tris MOPS gels (Thermo Fisher Scientific) and subsequently transferred onto nitrocellulose membranes. Blots were probed with antibodies to PIWIL1 (1:300; Abcam, ab12337), PIWIL2 (1:1000; Abcam, ab181340), PIWIL4 (1:1000; Abcam, ab180867), NF-κB p65 (1:1000; Cell Signaling Technology, 8242S), phospho-IκBα (Ser32) (1:1000; Cell Signaling Technology, 2859T), and β-actin (1:5000; Abcam) diluted in TBS-T containing 1% bovine serum albumin (BSA) overnight at 4°C. For phospho-antibodies, membranes were blocked with 5% BSA in TBS-T to prevent signal interference.

### Immunohistochemistry

Naive and tMCAo-operated mice were euthanized 24 h after the surgery, followed by cardiac perfusion using phosphate buffer (PB), followed by 4% of w/v paraformaldehyde in PB. Brains were rapidly removed on ice and postfixed overnight at 4°C and cryoprotected in 30% sucrose in 0.1 M PB with sodium azide 0.02% for 24 h at 4°C. Next, brains were sectioned frozen on a sliding cryostat at 20 μm thickness, in rostrum-caudal direction. Afterward, free-floating serial sections were incubated with PB Triton X 0.3% and blocking solution (0.5% milk, 10% FBS, and 1% BSA) for 1 h and 30 min. Immunostaining and confocal immunofluorescence procedures were performed as previously described.^35^ Following is a list of primary antibodies: PIWIL1 (rabbit polyclonal; 1:1000; abcam ab12337), PIWIL2 (rabbit polyclonal; 1:200; abcam ab181340), PIWIL4 (rabbit polyclonal; 1:200, abcam ab180867), anti-NeuN (mouse monoclonal; 1:1000; Millipore), NeuN (goat polyclonal antibody; 1:1000; Invitrogen; PA5-143586), anti-GFAP (mouse monoclonal; 1:500; Invitrogen 14-9892-82), and CD31 (1:300; mouse monoclonal; abcam ab24590).

### Profiling of chemokines

Blood was collected into EDTA-coated tubes prior to euthanizing the mice and stored at 4°C until processing. Samples were then centrifuged at 2,000 ×*g* for 10 min, and the plasma was used for chemokine profiling.

The levels of inflammatory mediators in plasma samples were measured in naive and tMCAo-operated mice at 6 and 24 h post-ischemia, as well as after ICV siRNA administrated followed by 24 h of ischemia. The levels were measured using LEGENDplex Multiplex Bead Assays (BioLegend, San Diego, CA). The Mouse Proinflammatory Chemokine Panel (cat. no. 740007) was used, which detects 13 chemokines: CCL2/MCP-1, CCL3/MIP-1α, CCL4/MIP-1β, CCL5/RANTES, CCL11/Eotaxin, CCL17/TARC, CCL20/MIP-3α, CCL22/MDC, CXCL1/KC, CXCL5/LIX, CXCL9/MIG, CXCL10/IP-10, and CXCL13/BLC. Chemokine profiling was performed on a BD FACSCanto II Cell Analyzer (BD Biosciences, San Jose, CA), and the FCS files generated were analyzed using BioLegend’s LEGENDplex Data Analysis Software, according to the manufacturer’s instructions.

### ChIP assay

All ChIP experiments were performed using ipsilateral cortical tissue collected from naive and tMCAo-operated mice at 24 h post-ischemia. Tissues were cross-linked using 1% formaldehyde, and the reaction was quenched with 0.125 M glycine. Samples were washed four times with ice-cold PBS containing protease inhibitors and then resuspended in 200 μL of lysis buffer (50 mM Tris, pH 8.1; 1% SDS; 10 mM EDTA; protease inhibitor cocktail). Chromatin was sheared to 200–500 bp fragments by using a Bandelin Sonopuls HD2070 ultrasonic homogenizer (Bandelin, Berlin, Germany), with three 15 s sonication cycles at 50% power. Chromatin lysates were incubated overnight with 3 μg of the following antibodies: NF-κB p65 (D14E12) (8242; Cell Signaling) and RelB (D7D7W) (10544; Cell Signaling). Normal rabbit IgG (sc-2357, Santa Cruz Biotechnology) served as negative controls. Following immunoprecipitation, DNA-protein complexes were captured by using 40 μL of salmon sperm DNA protein A/G agarose beads (16–157 and 16–201, Merck Millipore) for 2 h and subsequently processed as previously described.^32^ ChIP-enriched DNA was analyzed by real-time PCR using Fast SYBR Green Master Mix (ThermoFisher, 4385612). The PCR protocol consisted of an initial incubation at 50°C for 2 min, denaturation at 95°C for 10 min, followed by 35 amplification cycles (30 s at 95°C, 1 min at 60°C) with fluorescence detection at each cycle. Amplification of immunoprecipitated DNA was carried out using the following couples of primers:

(A) fw CHIP (m) CCL3: CTCAGCTTCTCTAACCACATGGA;

(A) rv CHIP (m) CCL3: GACCGCGTCTTCCCCATT;

(B) fw CHIP (m) CCL5: GCAGTTAGAGGCAGAGTCATAC;

(B) rv CHIP (m) CCL5: CCAGGGTAGCAGAGGAAGTG;

(C) fw CHIP (m) CXCL13: TGTAAAGGCAAGCTCAGCAATA;

(C) rv CHIP (m) CXCL13: CCTCCCACTGACCCTCTCTTT.

Results were normalized to the corresponding input DNA and expressed as a percentage relative to naive mice (Control) group.

### CoIP assay

Immunoprecipitation in lysates from ipsilateral cortical tissues was carried out as previously described.^36^ Briefly, 1 mg of total protein was incubated overnight at 4°C with 3 μg of the following antibodies: PIWIL2 (sc-377258; SantaCruz Biotechnology) and IKKα (26825; Cell Signaling). Samples immunoprecipitated with normal mouse or rabbit IgG were used as negative controls. Immune complexes were captured using 30 μL of Protein A/G PLUS-Agarose beads (sc-2003, Santa Cruz Biotechnology). The resulting immunoprecipitates were subsequently analyzed by western blotting.

### Statistical analysis

Statistical analyses were performed using GraphPad Prism 8 (GraphPad Software, San Diego, CA). The appropriate statistical test was selected based on sample size, group number, and variance homogeneity.

For datasets with unequal group sizes or heterogeneous variances including mRNA expression data (Figure 1), infarct volume measurements (Figure 6A), and plasma chemokine profiling (Figure 5), Brown-Forsythe and Welch ANOVA were employed, followed by Dunnett’s T3 post hoc test for pairwise comparisons. For protein expression data in Figure 1 and peripheral organ mRNA data (Figure 4), where group sizes and variances were homogeneous, ordinary one-way ANOVA was applied followed by Tukey’s post hoc test for multiple comparisons. For datasets with small or unequal sample sizes, including PIWIL2 knockdown validation and NF-κB pathway analyses (Figures 6B–6E), non-parametric Kruskal-Wallis tests were applied followed by Dunn’s post hoc test for multiple comparisons. For two-group comparisons in coIP and ChIP experiments (Figure 7), unpaired two-tailed Student’s *t* tests were applied. All data are presented as mean ± standard error of the mean (SEM), with individual data points representing independent biological replicates. A *p* value of <0.05 was considered statistically significant.

## Data and code availability

Data is available upon request to the corresponding authors.

## Acknowledgments

This work was supported by the following grants: CN00000041 “National Center for Gene Therapy and Drugs Based on RNA Technology” (concession number 1035 of 17 June 2022-PNRR MUR – M4C2 – Investment 1.4 Call “National Centers”, financed by EU- NextGenerationEU), code project (CUP) E63C22000940007 to G.P.; Next Generation EU, Mission 4, Component 2, PE0000006, CUP E63C2 2002170007 (Project “A multiscale integrated approach to the study of the nervous system in health and disease”, MNESYS), Spoke 3 (to G.P.); PRIN 2022, Project 2022NRT82A, “Short non-coding RNAs as mediators of brain adaptation to stroke: Exploitation as theranostic agents” to G.P.

## Author contributions

R.M.P., G.P., E.E., L.F., and L.A. conceived and designed the study. R.M.P., N.D.M., S.R., N.G., J.L., G.L., and L.C. developed the methodology. R.M.P., N.D.M., N.G., S.R., S.A., J.L., G.L., and L.C. performed experiments and acquired data. R.M.P., N.D.M., G.L., N.G., L.C., G.P., E.E., and L.A. analyzed and interpreted the data. R.M.P. and G.P. wrote the original draft of the manuscript. G.P., E.E., L.F., and L.A. supervised the study. All authors reviewed and approved the final manuscript.

## Declaration of interests

The authors declare no competing interests.

## Supplemental information

Document S1. Figures S1–S10

Document S2. Article plus supplemental information
