# Supplementary material for: PIWIL2 drives stroke progression via modulation of NF-κB signaling
Source: Mol Ther Nucleic Acids. 2026 Jul 24;37(3):103037. doi: 10.1016/j.omtn.2026.103037 (PMC13486804; doi:10.1016/j.omtn.2026.103037)
Supplement: Supplementary file 1 [file mmc1.pdf]

OMTN, Volume *37*

Supplemental information

PIWIL2 drives stroke progression via modulation of NF-κB signaling

Rohan Mahesh Patil, Giusy Laudati, Natascia Guida, Silvia Ruggiero, Noemi Di Muraglia, Serenella Anzilotti, Julian Lanthaler, Luigi Coppola, Luigi Formisano, Lucio Annunziato, Elga Esposito, and Giuseppe Pignataro

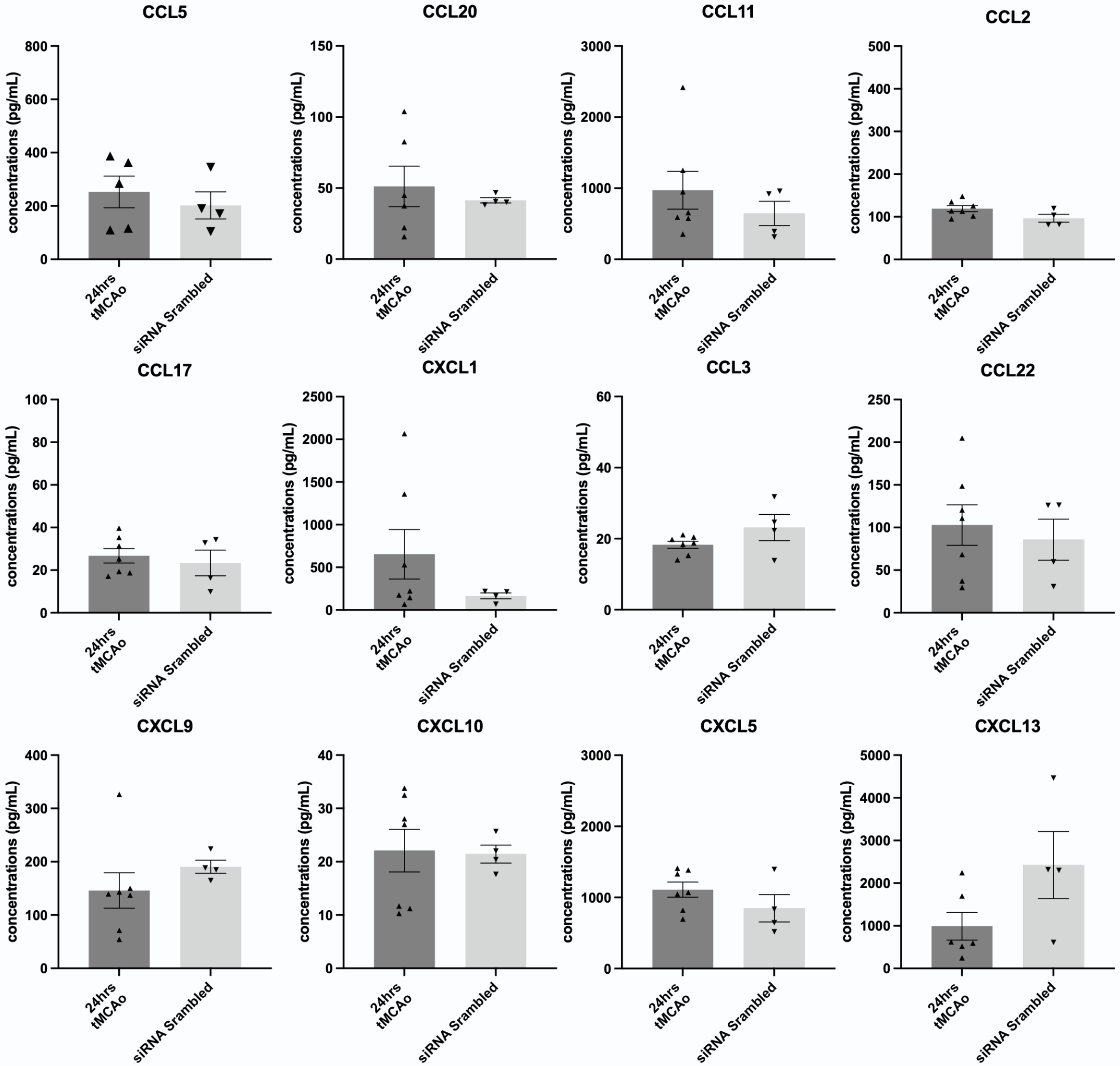

Figure S1 Cytokine and chemokine expression profiles following transient middle cerebral artery occlusion (tMCAO). Multiplex protein quantification (pg/mL) for chemokines and cytokines (CCL5, CCL20, CCL11, CCL2, CCL17, CXCL1, CCL3, CCL22, CXCL9, CXCL10, CXCL5, and CXCL13) in plasma comparing 24 h tMCAO vs. scrambled siRNA control conditions. Data points represent individual biological replicates, presented as mean +- SEM.

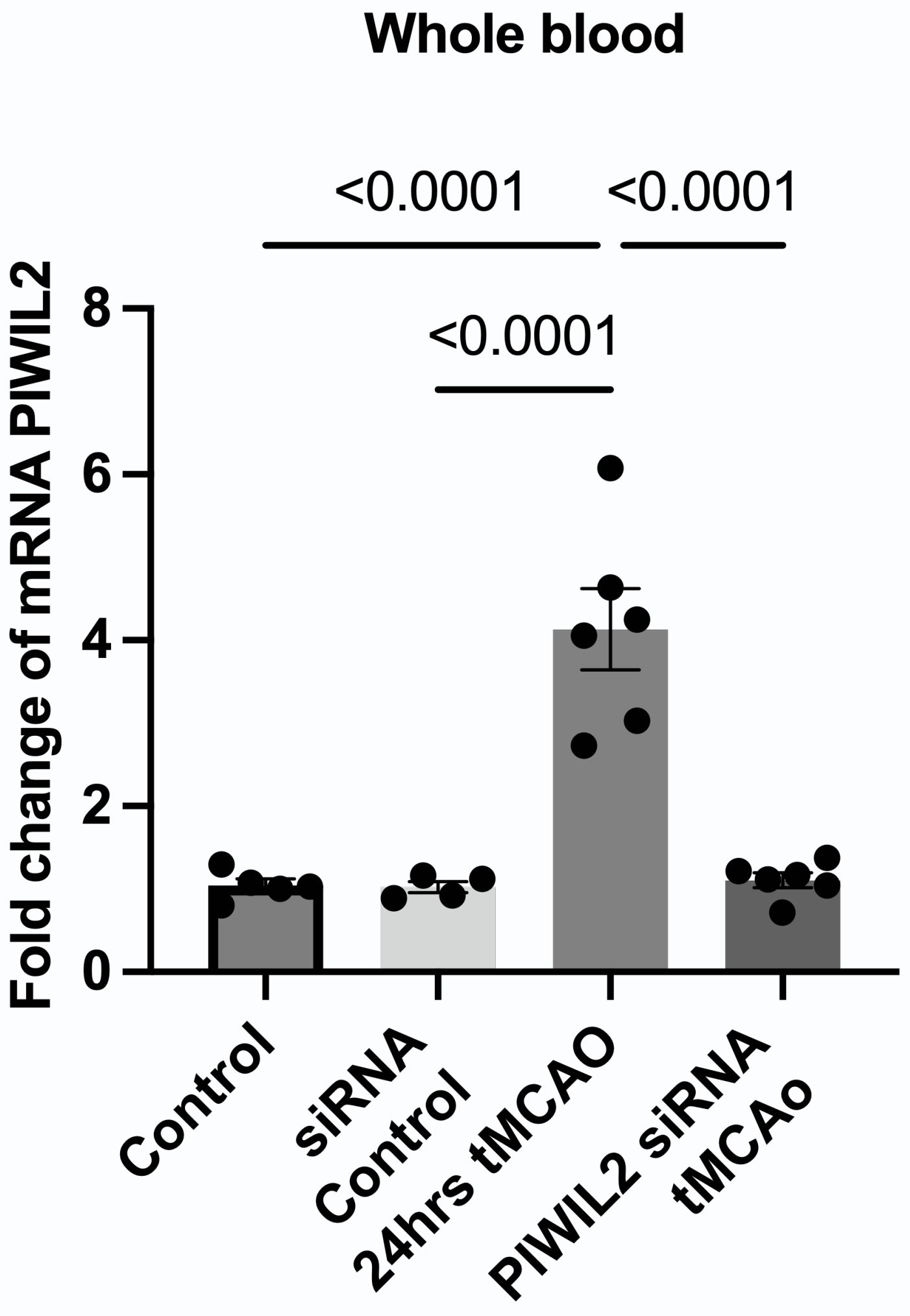

Figure S2 PIWIL2 mRNA expression levels in whole blood post-ischemia. Quantitative expression fold change of PIWIL2 mRNA measured in whole blood across Control, siRNA Control, 24hrs tMCAo, and PIWIL2 siRNA-treated 24hrs tMCAo groups. Individual data points represent independent biological replicates.

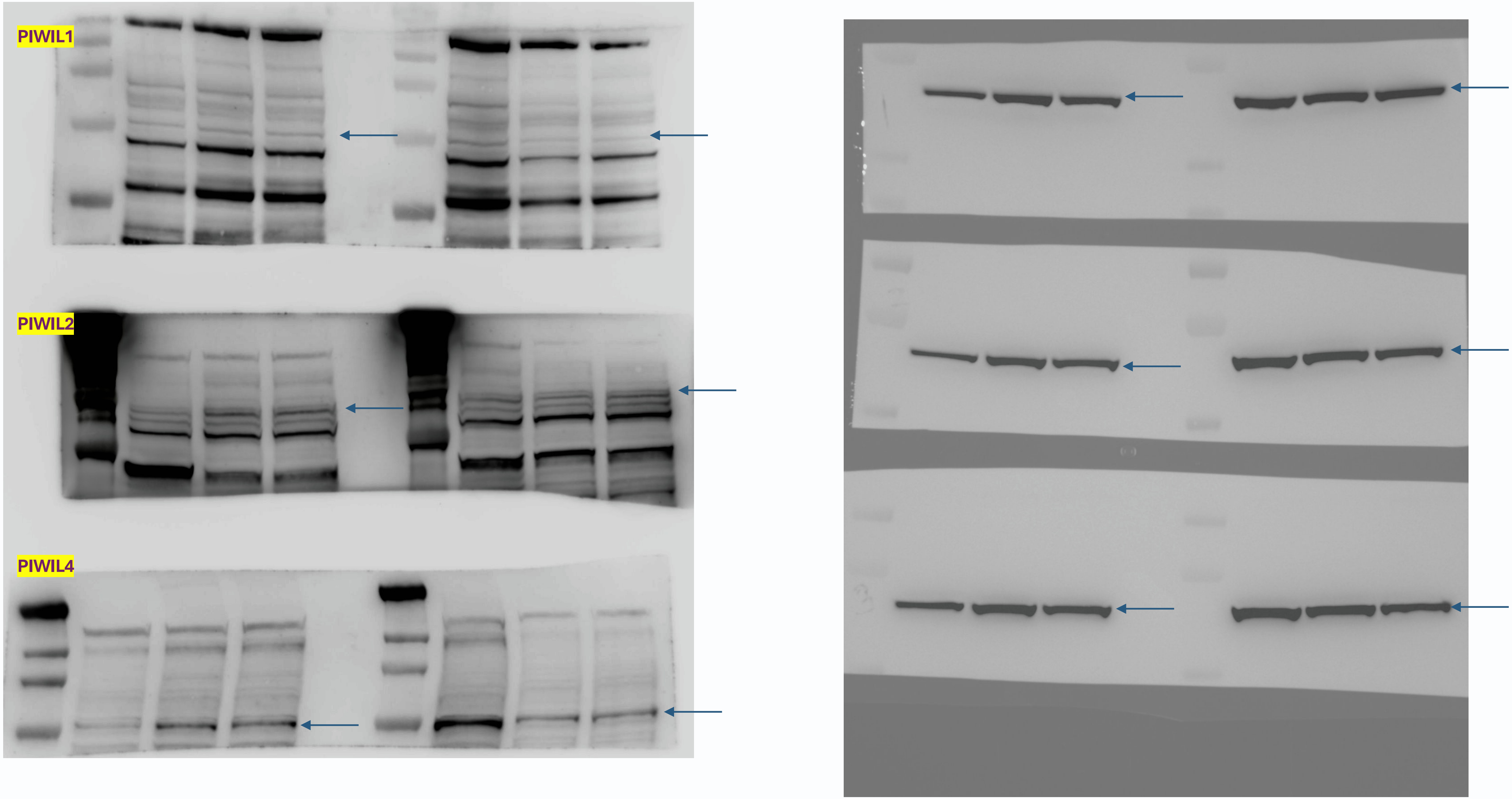

Figure S3 Complete uncropped immunoblot membranes showing target protein band detection for PIWIL1, PIWIL2, and PIWIL4 across experimental conditions. Blue arrows indicate the specific bands corresponding to each PIWIL protein isoform.

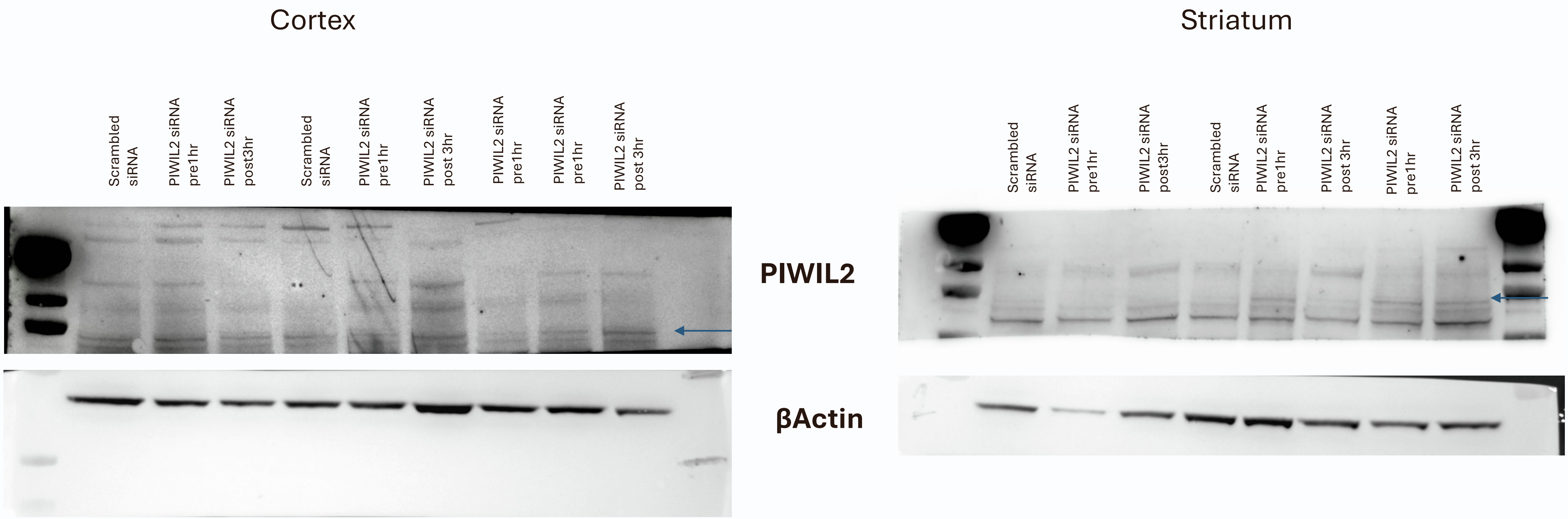

Figure S4 Uncropped Western blot membranes of PIWIL2 in cortical and striatal tissues. Full, uncropped immunoblots displaying PIWIL2 protein levels and corresponding loading control in βActin cortex and striatum tissue lysates. Treatment groups include Scrambled siRNA, PIWIL2 siRNA administered 1 h pre-ischemia (pre1hr), and PIWIL2 siRNA administered 3 h post-ischemia (post3hr). Blue arrows highlight specific PIWIL2 bands.

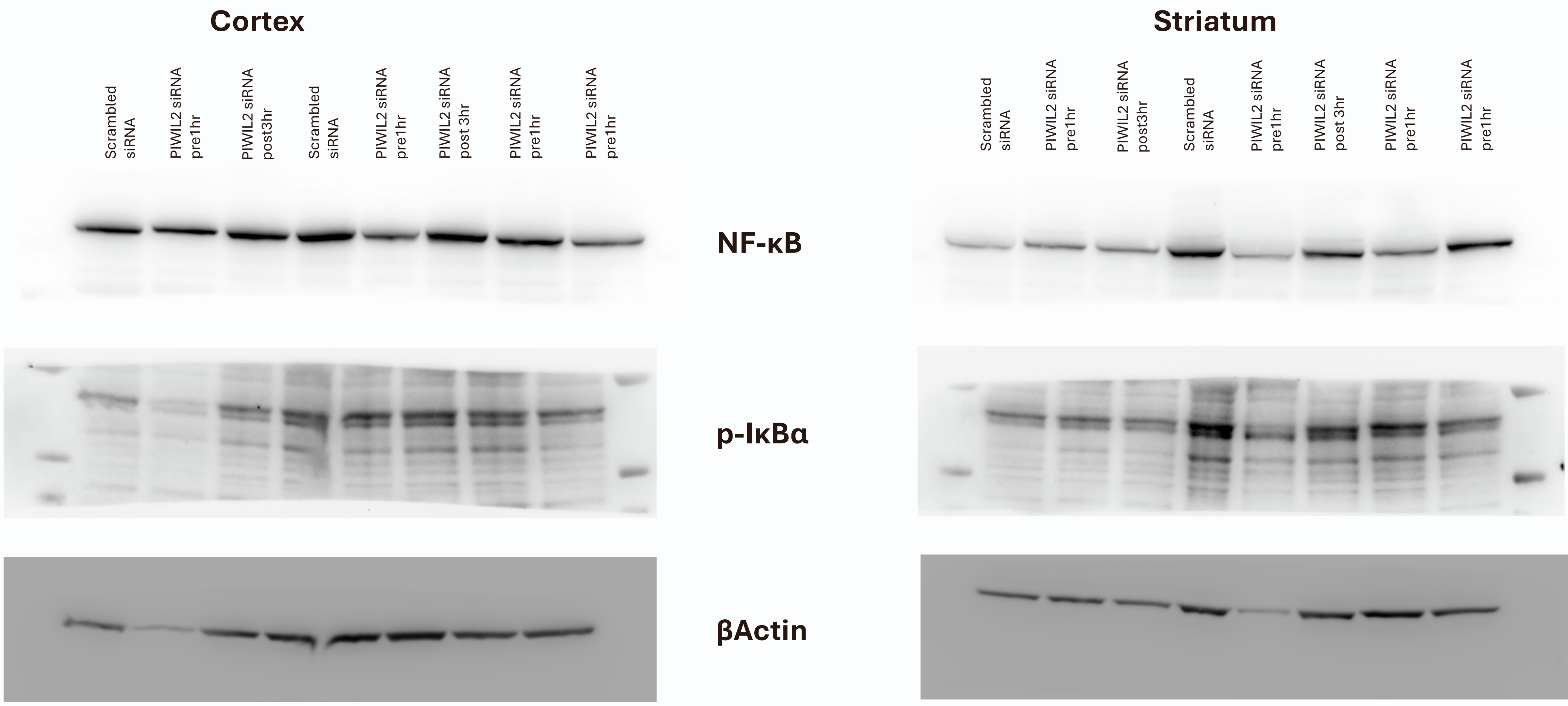

Figure S5 Uncropped Western blot membranes for NF-κB and p-IκBα signaling components. Complete, uncropped immunoblot membranes for total NF-κB, phosphorylated p-IκBα , and matching loading control βActin in cortex (left panels) and striatum (right panels) following treatment with Scrambled siRNA or PIWIL2 siRNA (pre-1 h and post-3 h).

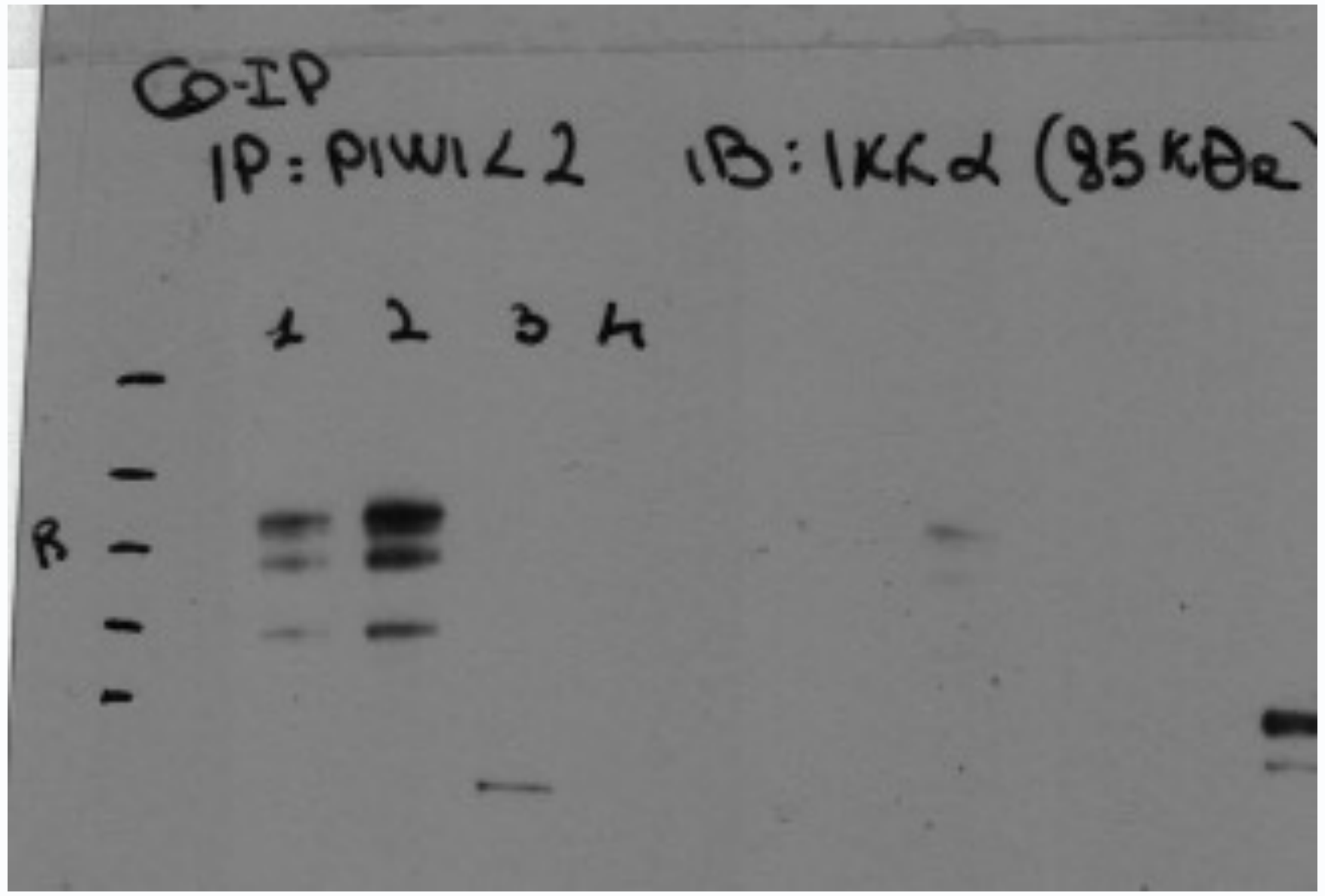

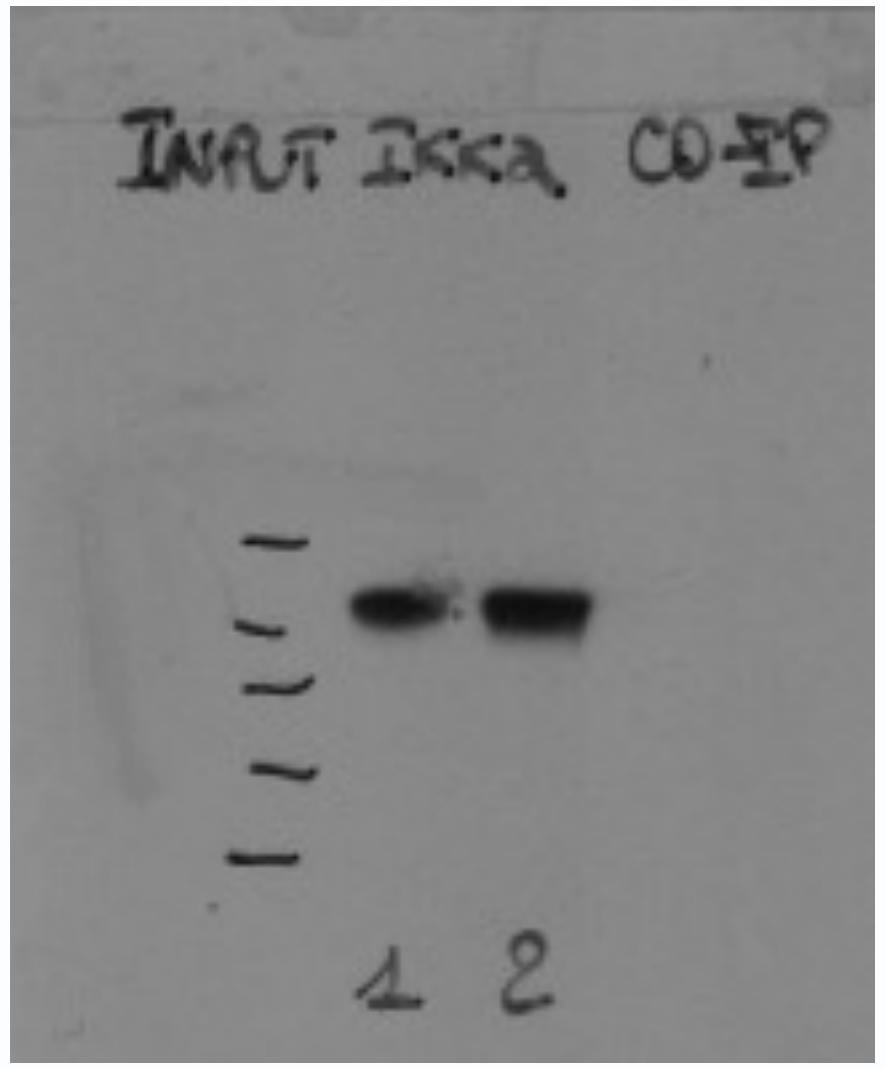

1) Naïve IP: PIWIL2
2) tMCAO 24h IP: PIWIL2
3) Naïve IP: IgG
4) tMCAO 24h IP: IgG

1) Naïve Input
2) tMCAO 24h Input

Figure S6 Uncropped Co-Immunoprecipitation (Co-IP) blot for PIWIL2 interaction with Iκκα Full immunoblot membrane demonstrating protein interaction via immunoprecipitation (IP) using anti-PIWIL2 antibody followed by immunoblotting (IB) with anti- **Iκκα** antibody (~85 kDa). Lanes represent: 1) Naïve IP: PIWIL2, 2) tMCAO 24h IP: PIWIL2, 3) Naïve IP: IgG control, 4) tMCAO 24h IP: IgG control, alongside total lysate input controls.

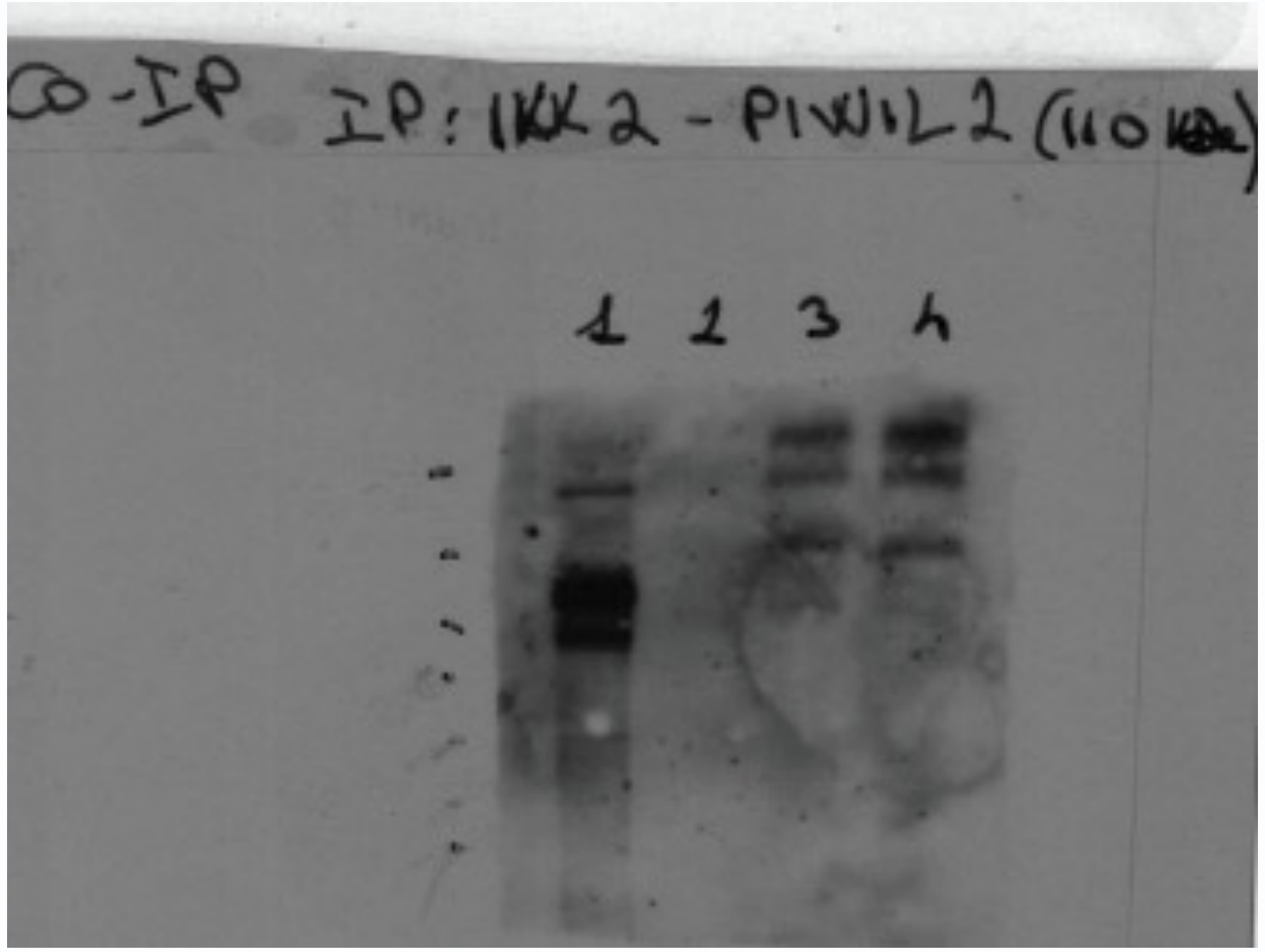

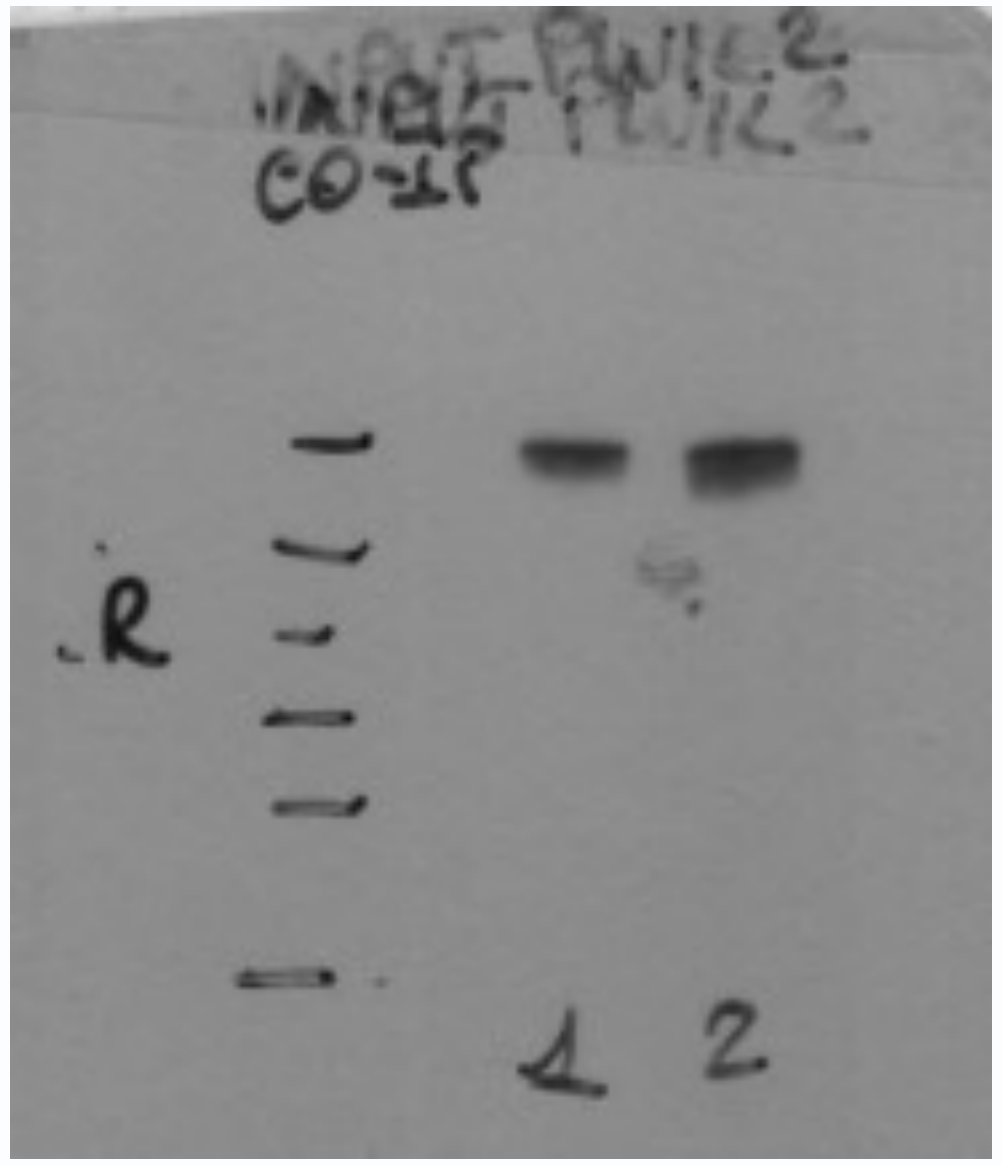

2) IP: IgG
3) Naive IP: IKKalpha
4) Tmcao 24h IP: IKKalpha

1) Naive Input
2) Tmcao 24h Input

Figure S7 Uncropped Co-Immunoprecipitation (Co-IP) blot for Iκκα interaction with PIWIL2. Reverse co-immunoprecipitation full blot membrane. Lysates were immunoprecipitated (IP) with anti- Iκκα antibody and immunoblotted (IB) with anti-PIWIL2 antibody (~110 kDa). Experimental conditions include Naïve, tMCAO 24h, IgG IP negative control, and total lysate inputs.

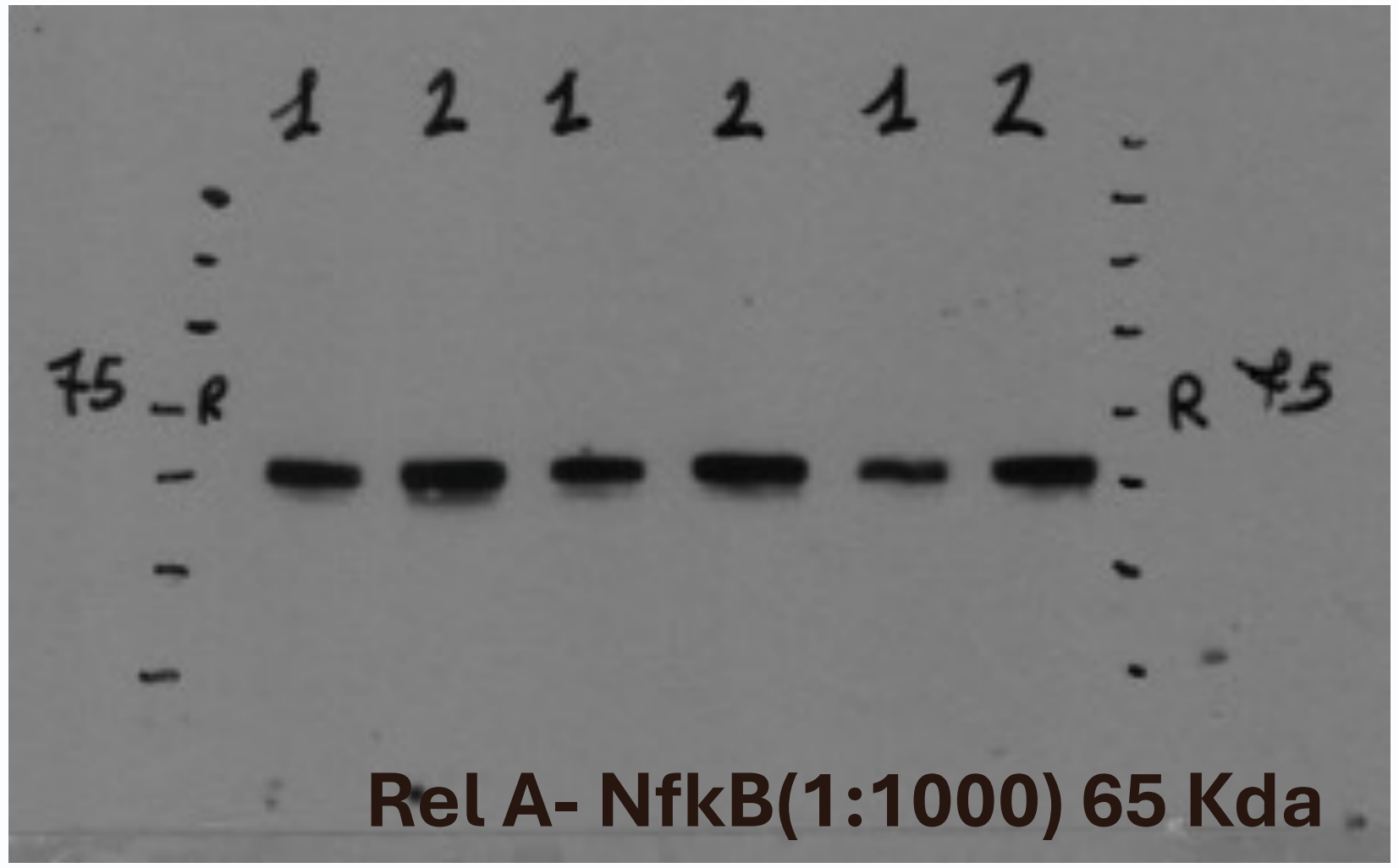

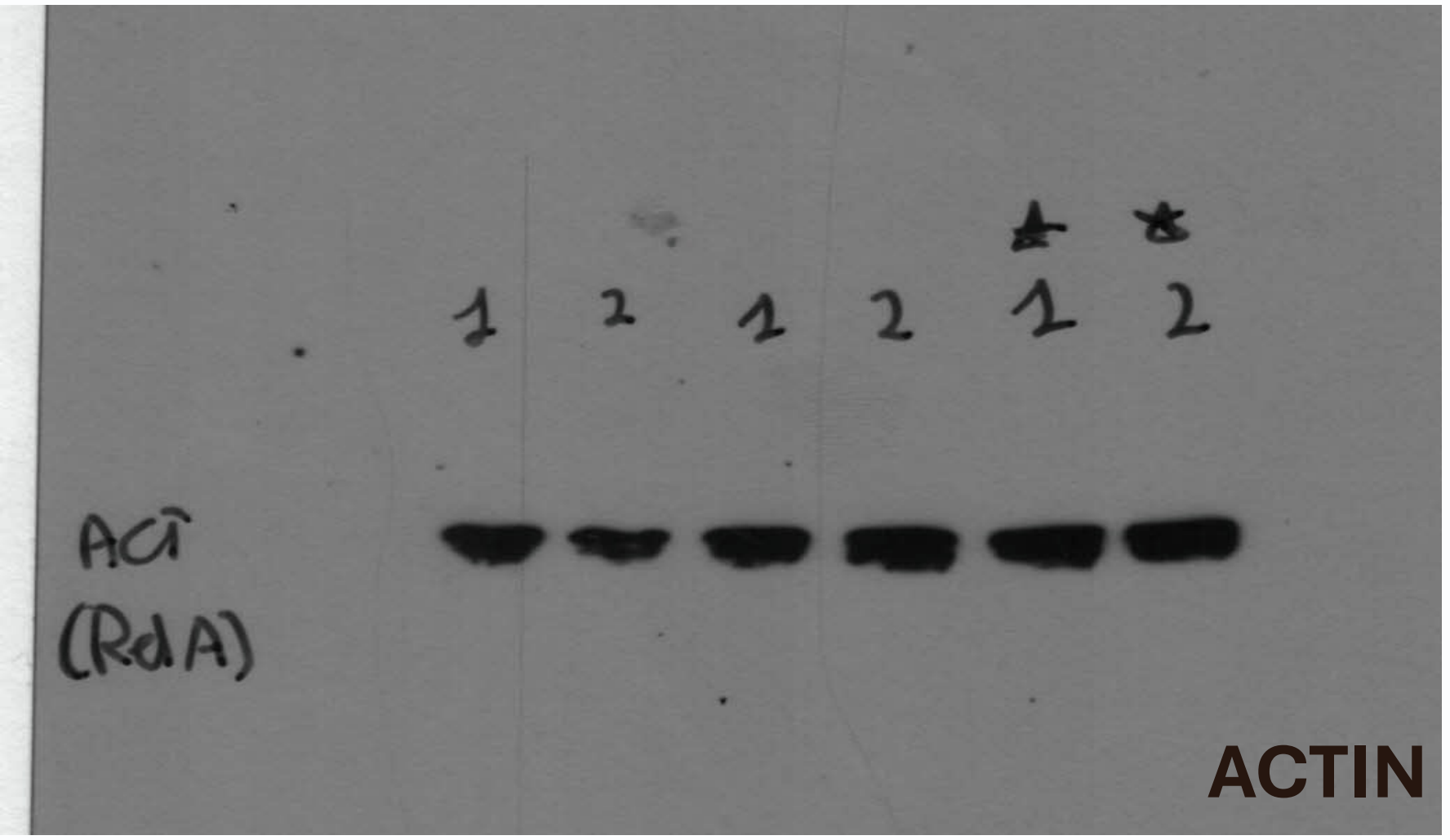

Rel A- NfkB(1:1000) 65 Kda
1) Naive
2) tMCAO 24h

Figure S8 Uncropped Western blot membranes for RelA (NF-κB) and Actin. Complete uncropped immunoblot membranes showing RelA/ NF-κB p65 subunit detection (primary antibody 1:1000, ~65 kDa band) and corresponding Actin loading control in Naïve (lane 1) and 24 h tMCAO (lane 2) brain tissue samples.

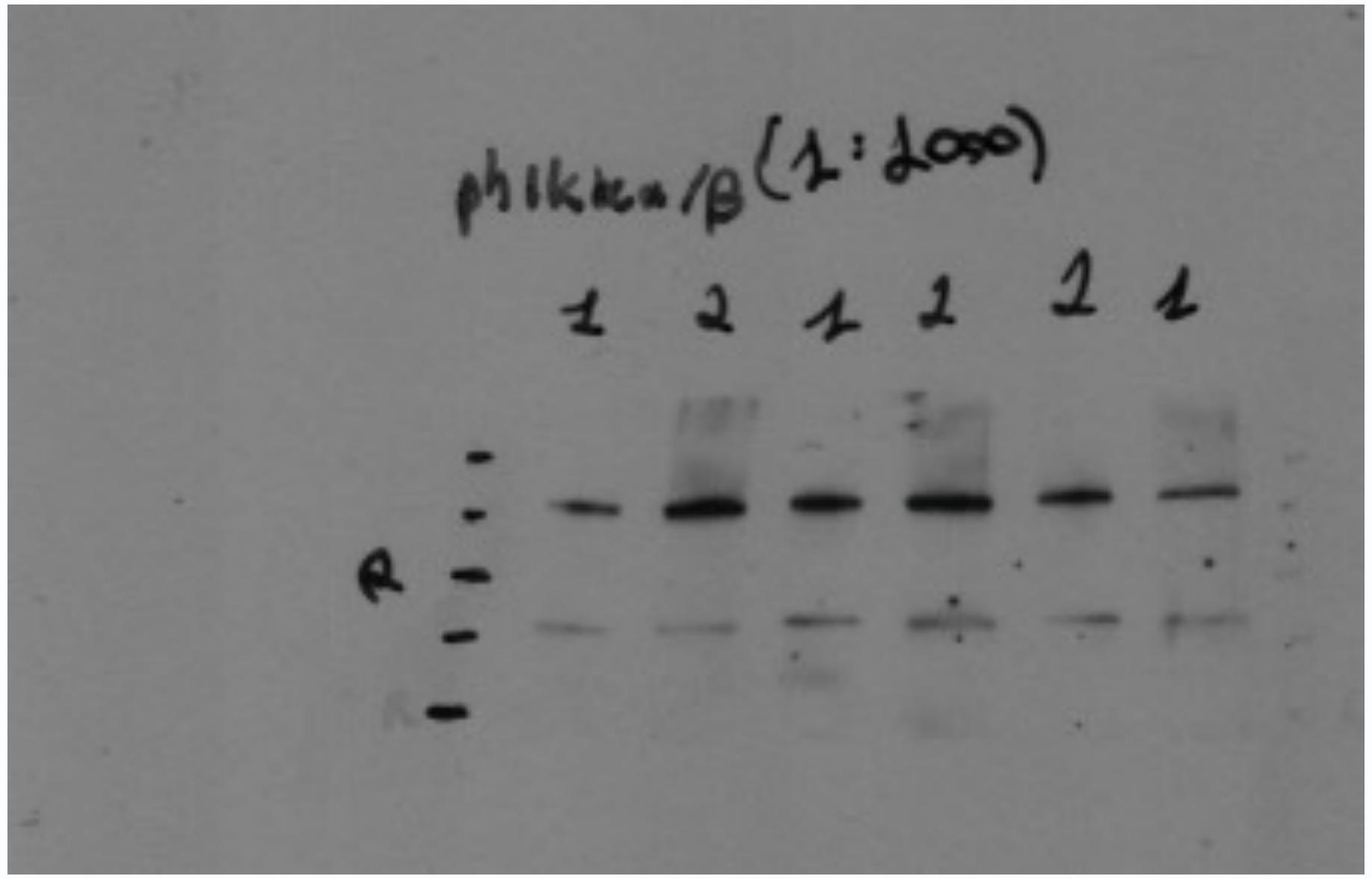

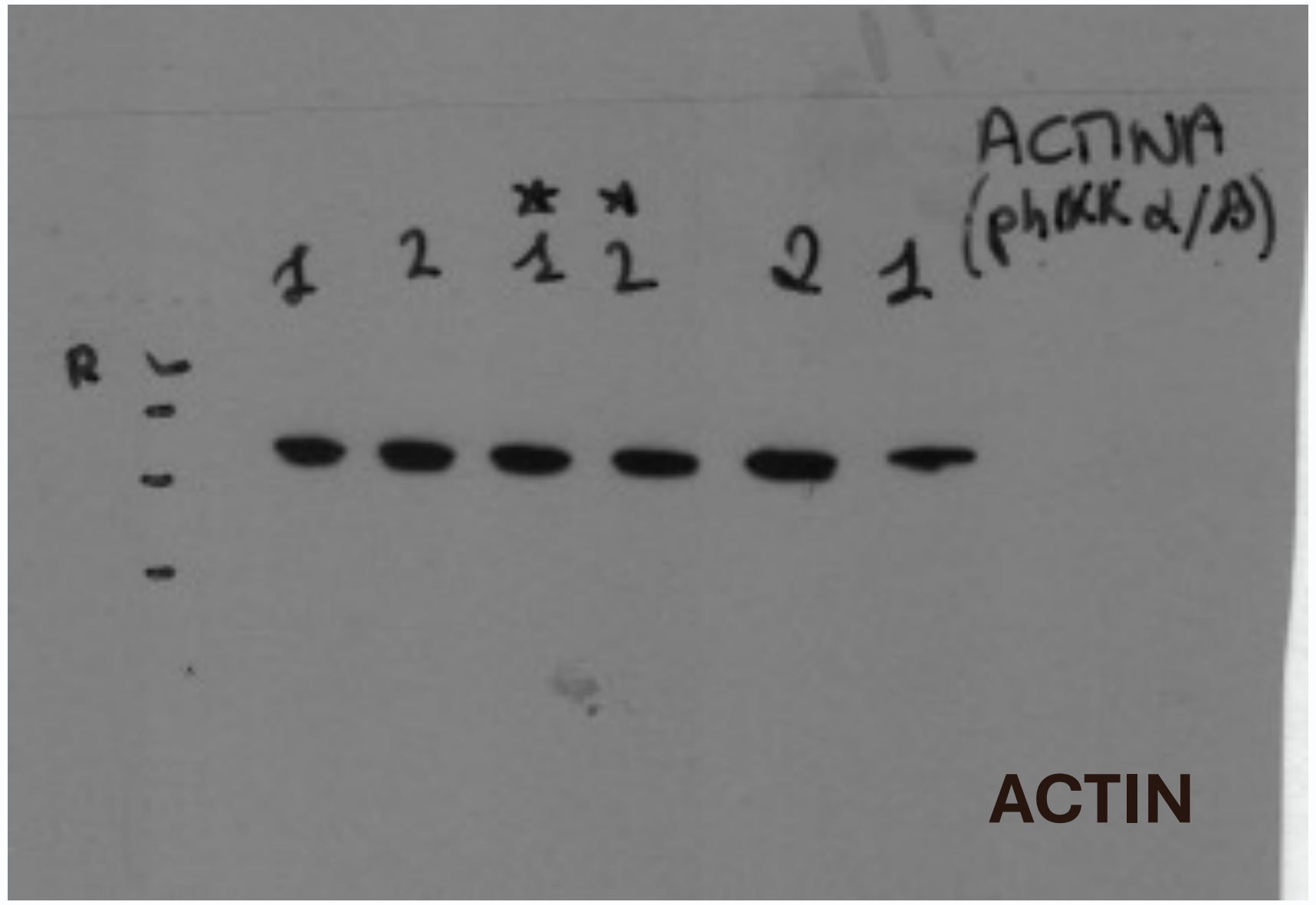

1) Naive
2) tMCAO 24h

Figure S9 Uncropped Western blot membranes for p-Iκκα/B and Actin. Full, uncropped immunoblot membranes for phosphorylated Iκκα/B (p-Iκκα/B, primary antibody 1:2000) and matching Actin loading control across Naïve (lane 1) and 24 h tMCAO (lane 2) tissue samples.

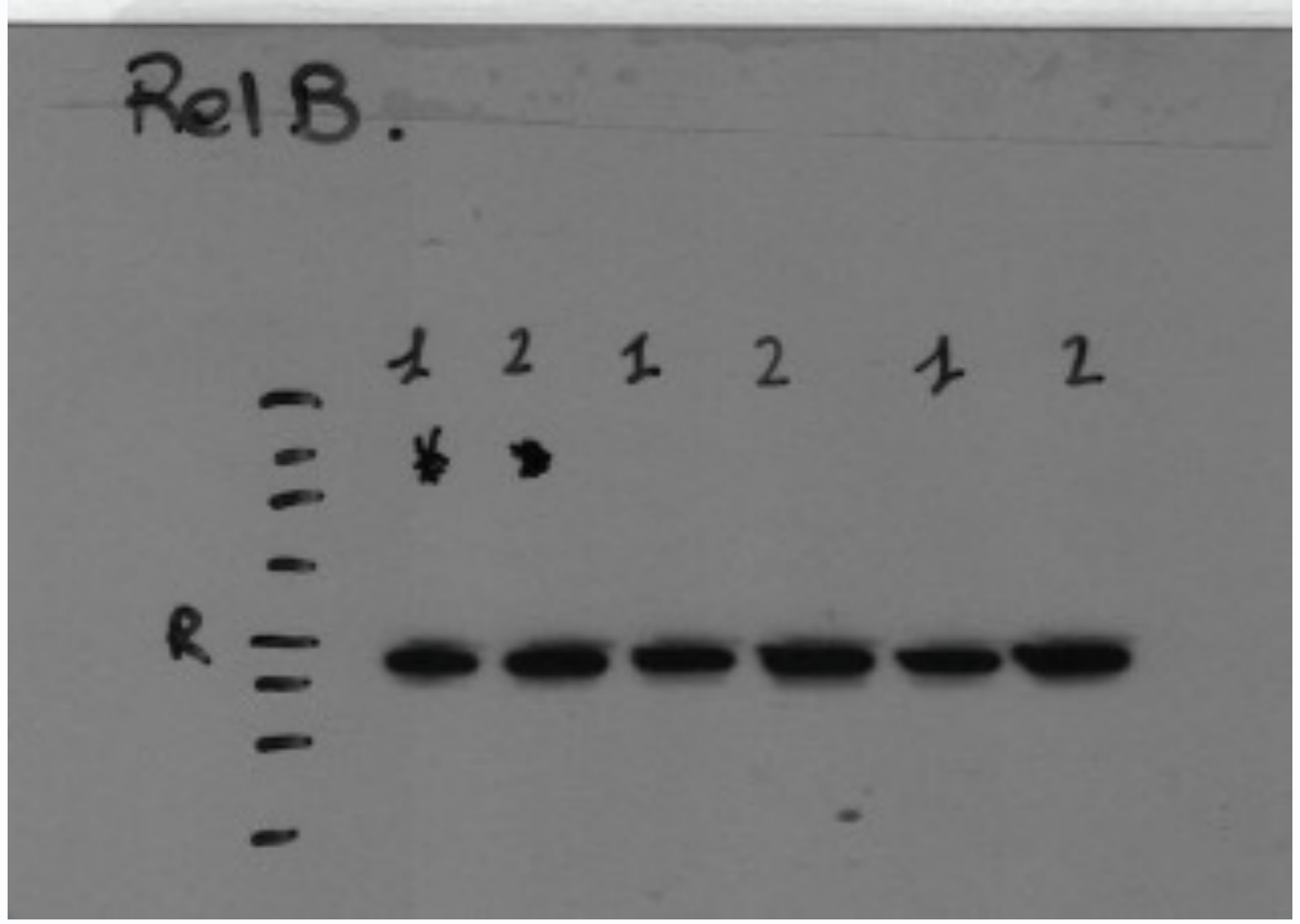

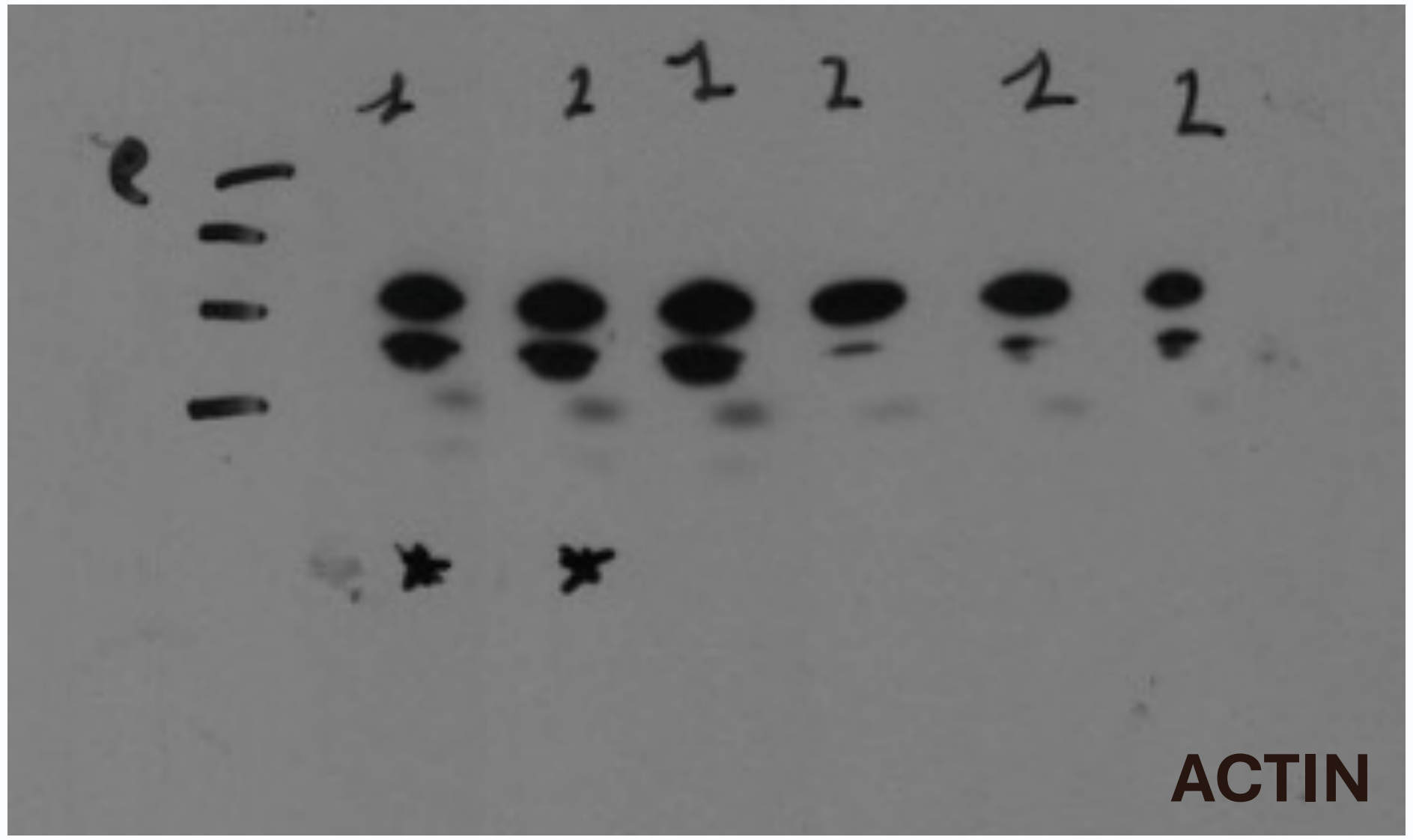

1) Naive
2) tMCAO 24h

Figure S10 Uncropped Western blot membranes for RelB and Actin. Complete uncropped immunoblot membranes for RelB protein expression and matching Actin loading control in Naïve (lane 1) and 24 h post-tMCAO (lane 2) tissue lysates.
